# A Deep Learning-Generated Mixed Tumor–Stroma Ratio for Prognostic Stratification and Multi-omics Profiling in Bladder Cancer

**DOI:** 10.34133/research.1053

**Published:** 2026-01-26

**Authors:** Yifeng He, Jinbo Xie, Suquan Zhong, Changxin Zhan, Fazhong Dai, Hongshen Lai, Mancun Wang, Yanyan He, Harsh Patel, Zhe-Sheng Chen, Biling Zhong, Xiaofu Qiu, Yadong Guo, Zongtai Zheng

**Affiliations:** ^1^ Department of Urology, The Affiliated Guangdong Second Provincial General Hospital of Jinan University, Guangzhou 510317, China.; ^2^ Department of Urology, The First Affiliated Hospital of Wannan Medical College (Yijishan Hospital of Wannan Medical College), Wuhu 241001, China.; ^3^ Department of Urology, Yuebei People’s Hospital Affiliated to Shantou University Medical College, Shaoguan 512026, China.; ^4^ Department of Urology, The People’s Hospital of Longhua, Shenzhen, Guangdong 518109, China.; ^5^ Department of Urology, People’s Hospital of Puning City, Puning 515300, China.; ^6^ Department of Pathology, The Affiliated Guangdong Second Provincial General Hospital of Jinan University, Guangzhou 510317, China.; ^7^ Department of Pharmaceutical Sciences, College of Pharmacy and Health Sciences, St. John’s University, New York, NY 11439, USA.; ^8^ Department of Pathology, School of Medicine, Shanghai Tenth People’s Hospital, Tongji University, Shanghai 200072, China.; ^9^ Department of Urology, Shanghai Tenth People’s Hospital, Tongji University, Shanghai 200040, China.

## Abstract

**Background:** Quantifying tumor–stroma architecture on routine hematoxylin and eosin slides may refine risk stratification in bladder cancer (BCa). We developed a convolutional neural network to segment whole-slide images, compute the mixed tumor–stroma ratio (MTSR), evaluate its prognostic value across multicenter cohorts, explore underlying molecular programs through multi-omics analysis, and construct a preoperative multiparametric MRI (mpMRI) radiomics model to estimate MTSR noninvasively. **Methods:** The ResNet50 convolutional network was customized using The Cancer Genome Atlas BCa slides labeled into 9 histological classes and background, followed by internal validation and multicenter external testing. Whole-slide-image-level segmentation yielded quantitative tissue ratios. The prognostic value was evaluated using Cox regression, Kaplan–Meier analysis, and meta-analysis, with a nomogram constructed by incorporating independent predictors. Prognostic significance was assessed by Cox regression, Kaplan–Meier analysis, and meta-analysis, and a nomogram was developed by integrating independent predictors. Bulk RNA sequencing underwent gene set variation analysis/gene set enrichment analysis, immune deconvolution, and ESTIMATE analyses, while single-cell RNA sequencing of high- vs. low-MTSR tumors profiled cellular heterogeneity, pseudotime trajectories, and regulon activity using SCENIC. An mpMRI-based random forest radiomics model was trained to predict high vs. low MTSR. **Results:** The convolutional neural network achieved >90% classification accuracy with Cohen’s kappa >0.95 in all cohorts. A nomogram combining MTSR and N stage outperformed clinicopathological predictors. Molecular analyses revealed that high-MTSR tumors displayed increased macrophage infiltration and enrichment of pathways related to extracellular matrix remodeling, cell adhesion, and transforming growth factor-β/WNT signaling. Single-cell analysis identified an integrin subunit beta 8 (ITGB8)-high urothelial subtype (cluster 8) with terminal differentiation, enhanced WNT activity, and sender-dominant communication networks. The mpMRI radiomics model achieved accuracies of 0.701 and 0.710 for predicting MTSR status in the training and validation sets, respectively. **Conclusions:** The deep learning-generated MTSR showed consistent reproducibility and prognostic independence across cohorts, mechanistically connected with an ITGB8-enriched stromal–oncogenic pathway. Its estimation via mpMRI radiomics enables integrative, noninvasive risk stratification for precision management of BCa.

## Introduction

Bladder cancer (BCa) represents a common malignancy of the urinary system, posing persistent clinical challenges due to its high recurrence and progression tendencies [1]. Clinically, BCa is classified into non-muscle-invasive bladder cancer (NMIBC), which represents approximately 75% of cases and is limited to the urothelial mucosa (Ta) or lamina propria (T1), and muscle-invasive bladder cancer (MIBC), characterized by detrusor muscle infiltration (T2) or extension beyond the muscularis (T3 and T4) [2].

Pathological examination is essential in determining the stage and grade of BCa, which directly influences treatment decisions and prognosis [3]. Hematoxylin and eosin (H&E) staining is a standard technique used in pathology to visualize tissue architecture and cellular details. However, the traditional approach to pathological diagnosis depends on the manual review of H&E-stained slides by pathologists, a process that is time-consuming and prone to interobserver variability. In addition, while the tumor–node–metastasis (TNM) staging system provides a foundational framework for determining treatment strategies in BCa, the variability in patient outcomes within the same stage underscores the need for more precise and informative prognostic markers [4–6].

Currently, digital pathology is emerging as a transformative technology in the field of pathology [7]. By converting conventional glass slides into whole-slide images (WSIs), through digital pathology, histopathological data can be efficiently stored, exchanged, and analyzed with improved standardization and reproducibility [8]. This technological advancement has paved the way for the application of artificial intelligence (AI) in pathology. AI has shown great promise in automating the analysis of large-scale medical data [9–12], especially histopathological images [13,14]. Through the use of deep learning models, AI can learn to recognize and classify different tissue types, detect subtle histological features, and even predict clinical outcomes [15–17]. Prior BCa studies have shown promise, but most have been limited by small datasets, narrow label definitions, lack of external validation, or outcome scores that are difficult to interpret biologically [18–20]. In parallel, preoperative multiparametric MRI (mpMRI) is widely used in BCa. By transforming MRI data into quantitative features, radiomics provides detailed information about tissue structure, texture, and shape, often beyond what traditional imaging can reveal [21]. These quantitative features are valuable for supporting pathological and clinical decision-making, especially in predicting tumor histological states, subtypes, prognosis, and treatment responses [22–26].

**Fig. 1.**
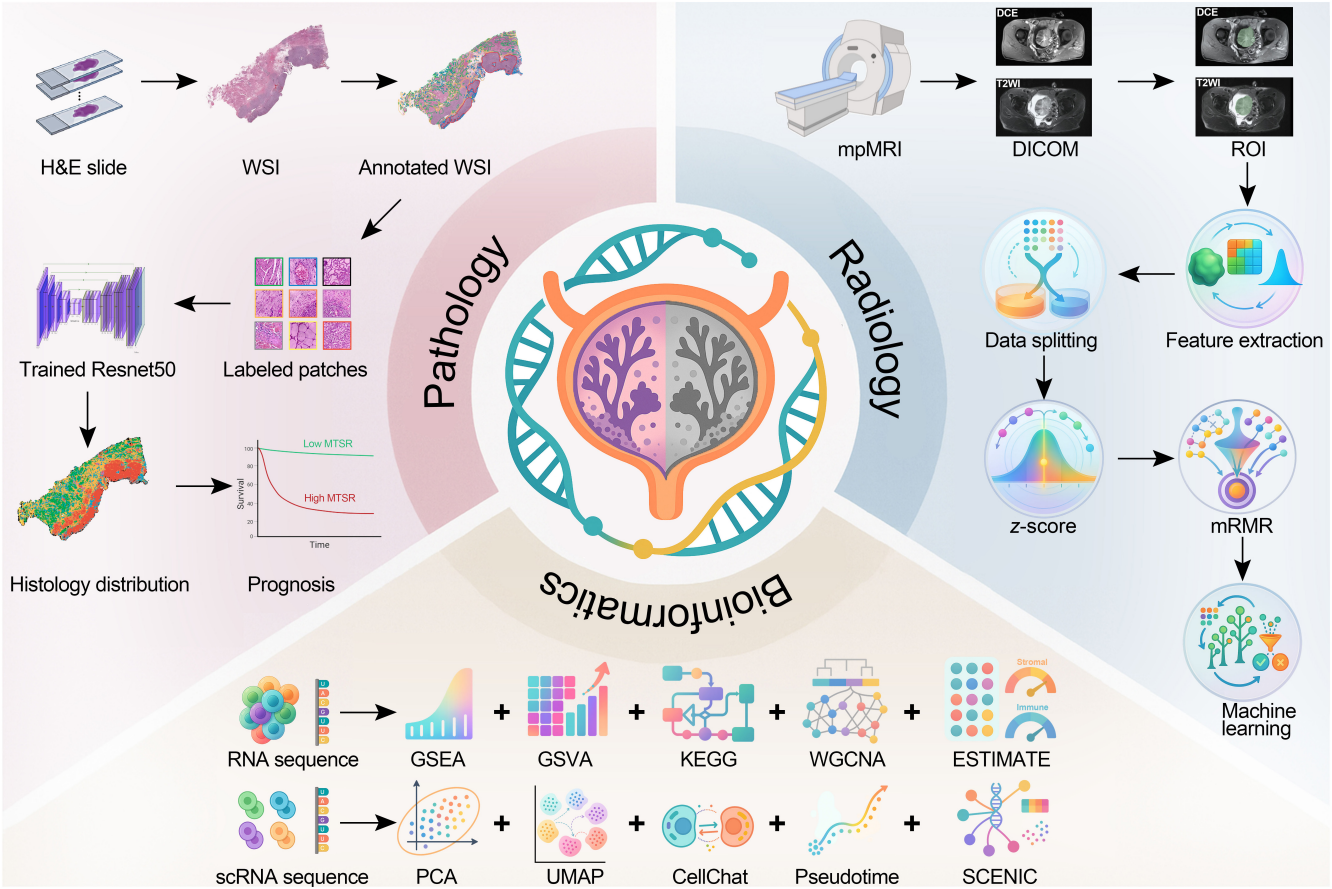
Workflow of this study. H&E, hematoxylin and eosin; WSI, whole-slide image; MTSR, mixed tumor–stroma ratio; mpMRI, multiparametric MRI; DCE, dynamic contrast enhanced; T2WI, T2-weighted imaging; ROI, region of interest; mRMR, minimum redundancy maximum relevance; GSEA, gene set enrichment analysis; GSVA, gene set variation analysis; KEGG, Kyoto Encyclopedia of Genes and Genomes; WGCNA, weighted gene co-expression network analysis; scRNA, single-cell RNA; PCA, principal component analysis; UMAP, uniform manifold approximation and projection.

We aim to construct a convolutional neural network (CNN) model for fully automated analysis of H&E-stained WSIs in BCa (shown in Fig. 1). The model is designed to mitigate or eliminate interobserver variability in tissue type classification and prognostic assessment. Through the automated identification, quantification, and visualization of histological subtypes across multicenter datasets, we rigorously validated the model’s accuracy in tissue recognition and assess the prognostic value of histological subtype proportions, especially the mixed tumor–stroma ratio (MTSR). In addition, we integrated bulk RNA sequencing (RNA-seq), pathway enrichment, immune deconvolution, and single-cell RNA sequencing (scRNA-seq) to characterize molecular programs and cell states associated with high versus low MTSR. Finally, we explored translation to the preoperative setting by constructing an mpMRI-based radiomics model to predict MTSR noninvasively.

## Results

### Patients

Figure S1 provides a detailed overview of the inclusion and exclusion criteria applied to patients across the various datasets. A total of 379 BCa patients (450 WSIs) from The Cancer Genome Atlas (TCGA) dataset were included to explore the prognostic value of the tissue class ratios and their relationship with pathological characteristics. In the external validation sets, a total of 1,053 BCa patients (1,271 WSIs) were included to validate the prognostic value of the tissue class ratios (Table 1). Age and sex information was extracted directly from patients’ identification cards as recorded in medical records. Sex was documented as male or female based on the identification card.

**Table 1.** Baseline characteristics of bladder cancer patients from multiple datasets

Characteristics	Number (%)					
	TCGA (*n* = 379)	GD2H (*n* = 179)	STPH (*n* = 168)	YJSH (*n* = 156)	ZSSY (*n* = 251)	PN (*n* = 112)
Sex						
Men	282 (74.41)	154 (86.03)	137 (81.55)	122 (78.21)	203 (80.88)	99 (88.39)
Women	97 (25.59)	25 (13.97)	31 (18.45)	34 (21.79)	48 (19.12)	13 (11.61)
Age (years)						
<65	137 (36.15)	19 (10.61)	52 (30.95)	53 (33.97)	130 (51.79)	33 (29.46)
≥65	242 (63.85)	160 (89.39)	116 (69.05)	103 (66.03)	121 (48.21)	79 (70.54)
Pathologic T stage						
<pT2	3 (0.79)	125 (69.83)	114 (67.86)	101 (64.74)	46 (18.33)	14 (12.50)
≥pT2	376 (99.21)	54 (30.17)	54 (32.14)	55 (35.26)	205 (81.67)	98 (87.50)
Pathological grade						
Low	21 (5.54)	68 (37.99)	34 (20.24)	61 (39.10)	134 (53.39)	66 (58.93)
High	355 (93.67)	111 (62.01)	134 (79.76)	95 (60.90)	117 (46.61)	46 (41.07)
NA	3 (0.79)	0	0	0	0	0

TCGA, The Cancer Genome Atlas; GD2H, Affiliated Guangdong Second Provincial General Hospital of Jinan University; STPH, Shanghai Tenth People’s Hospital; YJSH, Yijishan Hospital; ZSSY, Third Affiliated Hospital of Sun Yat-Sen University; PN, People’s Hospital of Puning City; pT2, pathologic tumor stage 2; NA, not available

### Performance of the neural network

**Fig. 2.**
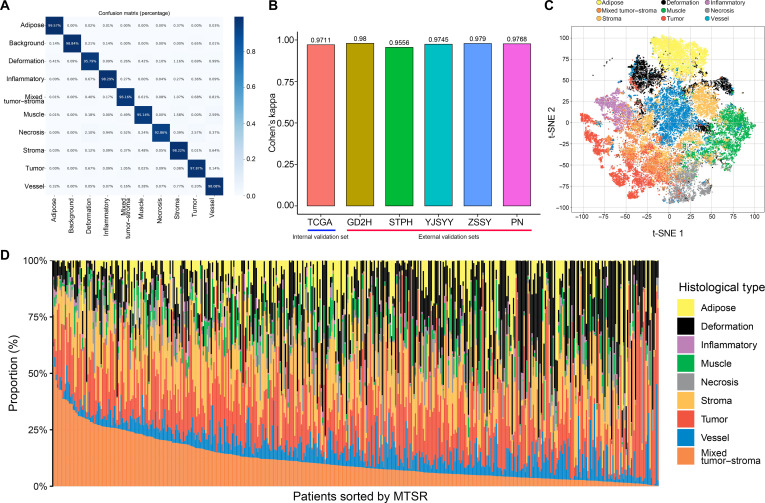
Performance of the trained ResNet50 model in tissue classification. (A) Confusion matrix displaying the classification accuracy of the trained ResNet50 model for the background patches and 9 tissue classes on the internal validation dataset from TCGA. (B) Cohen’s kappa values of the trained ResNet50 model in each validation dataset. (C) t-distributed stochastic neighbor embedding (t-SNE) visualization of the deep learning features extracted from the final average pooling layer of the trained ResNet50 model. (D) Bar graph showing the distribution of the 9 histological tissue types across the H&E-stained WSIs in the TCGA dataset.

Figures S2 and S3A illustrate the distribution and representative images of each tissue class across the training set, the TCGA internal validation cohort, and 5 external validation cohorts. The ResNet50 network developed in this study exhibited strong discriminative capability for all 10 tissue categories. The classification accuracy for each category exceeded 90% in both the internal validation dataset (Fig. 2A) and the 5 independent external datasets (Fig. S3B to F). Correspondingly, Cohen’s *κ* coefficients were consistently above 0.95 (Fig. 2B), confirming excellent model reliability. Moreover, t-distributed stochastic neighbor embedding (t-SNE) visualization of the high-dimensional features extracted from the trained ResNet50 model demonstrated that samples belonging to different tissue categories formed clearly separated clusters in the embedding space (Fig. 2C).

### Evaluation of the prognostic value of tissue class ratios in TCGA

**Fig. 3.**
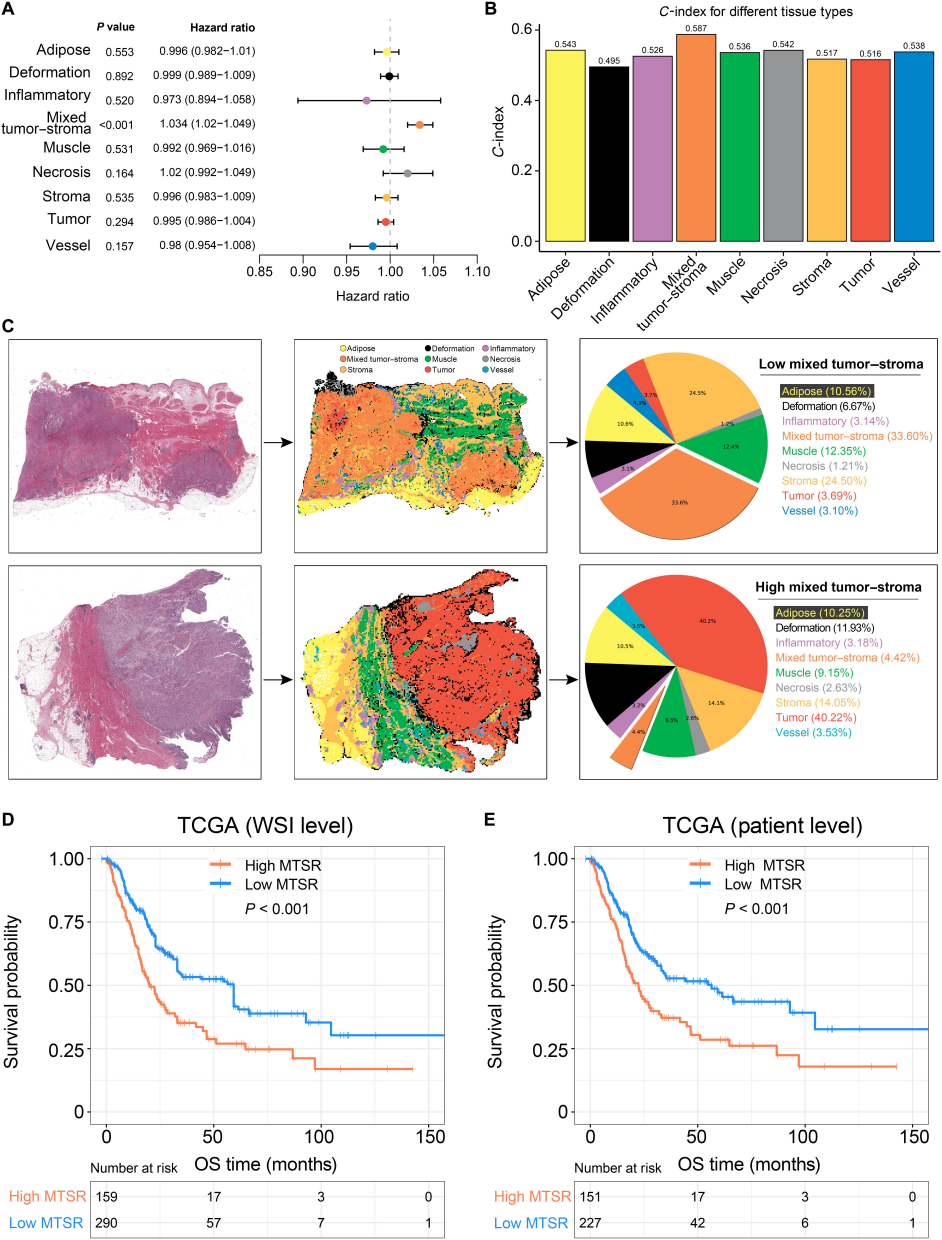
Prognostic significance of MTSR in the TCGA dataset. (A) Univariate Cox proportional hazards regression analysis of the tissue class ratios. (B) Concordance index (*C*-index) values of the prognostic models based on each tissue class ratio. (C) Representative examples of WSIs with high and low MTSRs as identified by the convolutional neural network (CNN) model. (D) Kaplan–Meier survival curves for overall survival (OS) at the WSI level. (E) Kaplan–Meier survival curves for OS at the patient level.

The distribution of histological subtypes across H&E-stained WSIs was examined, and the relative proportion of each subtype within the TCGA cohort was visualized (Fig. 2D). The MTSR exhibited a wide range, varying from 0.16% to 61.9%. In the TCGA BCa dataset, univariate Cox regression assessing the proportions of the 9 histological categories identified MTSR as the only subtype significantly associated with prognosis (Fig. 3A; hazard ratio [HR] = 1.034, 95% confidence interval [CI]: 1.020 to 1.049). Moreover, MTSR achieved the highest prognostic concordance index among all categories (Fig. 3B; *C*-index = 0.587). Representative cases with high and low MTSRs predicted by the ResNet50-based model are displayed in Fig. 3C.

Kaplan–Meier survival analysis at the WSI level demonstrated that patients with elevated MTSR values experienced significantly shorter overall survival (OS) compared to those with a lower MTSR (Fig. 3D; *P* < 0.001). A consistent trend was observed when survival outcomes were analyzed at the patient level (Fig. 3E; *P* < 0.001), reinforcing the independent prognostic relevance of MTSR in BCa.

### Consistency analysis of MTSR

A high degree of consistency was observed between the ResNet50-derived tissue classifications and the expert pathologist’s manual annotations (Fig. S4A). Within the TCGA training cohort, the MTSR values estimated by the model showed a strong linear correlation with those obtained from pathologist assessment (Pearson’s *r* = 0.917, *P* < 0.001).

Agreement analysis further demonstrated excellent concordance between the manual and model-derived MTSR measurements, as indicated by a 2-way random-effects intraclass correlation coefficient (ICC = 0.90; 95% CI: 0.849 to 0.935). Consistent findings were supported by the Bland–Altman analysis (Fig. S4B), which revealed minimal systematic bias between the 2 methods. The average difference between pathologist-annotated and model-predicted MTSR values was −0.013 (95% CI: −0.022 to −0.004), confirming a high level of reproducibility across evaluation approaches.

### Validation of the prognostic value of MTSR

**Fig. 4.**
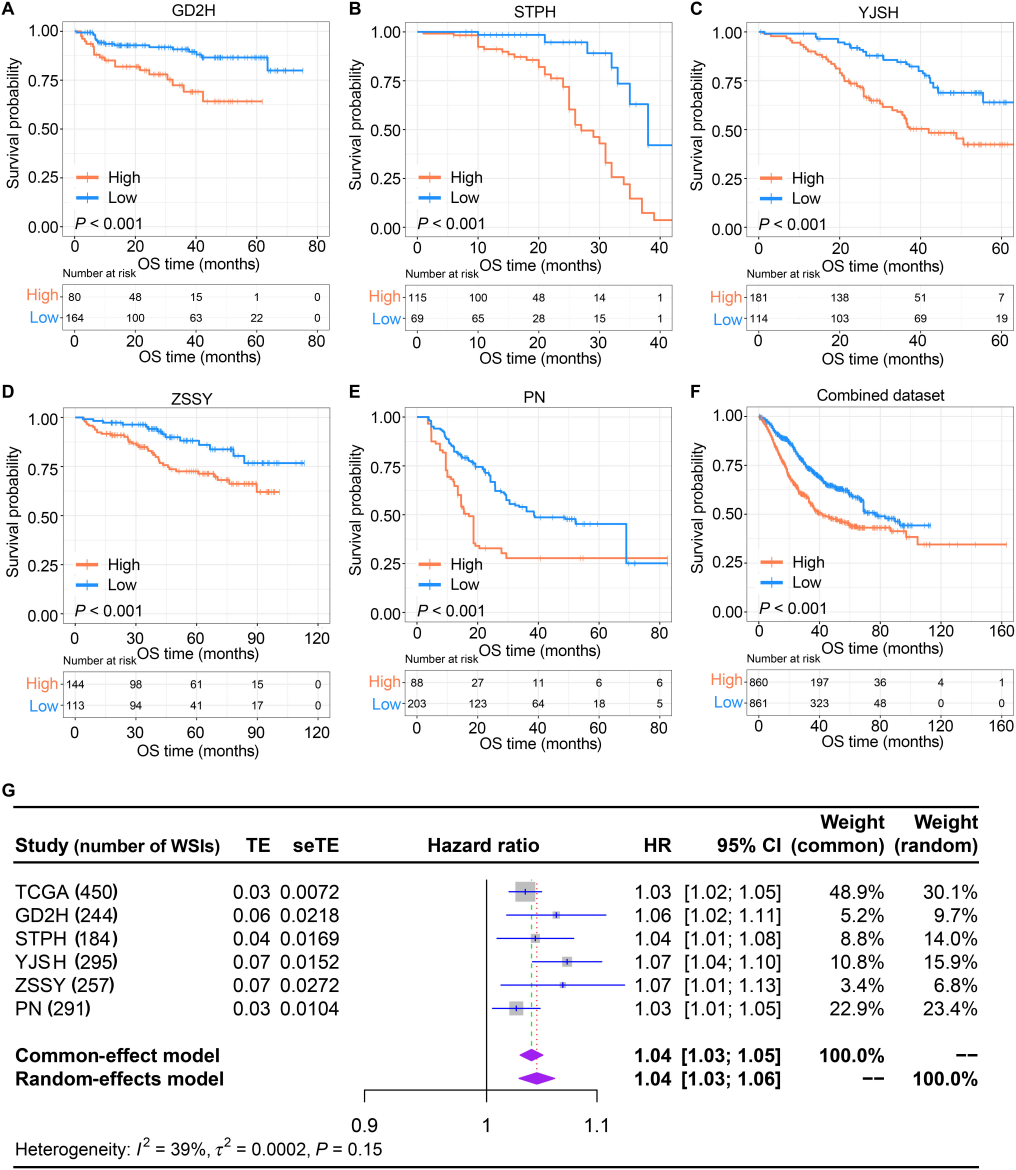
External validation of the prognostic value of MTSR in bladder cancer patients. (A to E) Kaplan–Meier survival curves for OS in the 5 external validation datasets (GD2H, STPH, YJSH, ZSSY, and PN), stratified by high- and low-MTSR groups. (F) Kaplan–Meier survival curve for OS in the combined external validation cohort (*n* = 1,271). (G) Forest plot of the meta-analysis of univariate Cox regression results for MTSR across the TCGA dataset and the 5 external validation datasets. TE, treatment effect; seTE, standard error of treatment effect; CI, confidence interval; HR, hazard ratio.

To further validate the prognostic value of MTSR, this study also examined the associated between MTSR and survival outcomes across 5 external validation datasets. High-MTSR patients had poorer OS compared to those with a low MTSR (Fig. 4A to E, all *P* < 0.001) in all 5 external datasets. After combining the patient data from these 5 datasets into a combined dataset and performing Kaplan–Meier analysis (using a cutoff value of the median 4.275%), it was found that a high MTSR was associated with worse OS in BCa patients (Fig. 4F). Further stratification into NMIBC and MIBC subgroups, using the respective MTSR median values for each group to distinguish low and high MTSRs, revealed that a high MTSR was linked to significantly worse OS in NMIBC (*P* < 0.001) and MIBC patients (*P* = 0.011) (Fig. S5). Finally, integrating univariate Cox estimates from TCGA and all 5 external cohorts under a fixed-effects meta-analysis yielded a pooled HR of 1.04 per unit increase in MTSR (95% CI, 1.03 to 1.05), underscoring its stability and utility as a prognostic indicator in BCa (Fig. 4G).

### Construction of a clinical prognostic model

**Fig. 5.**
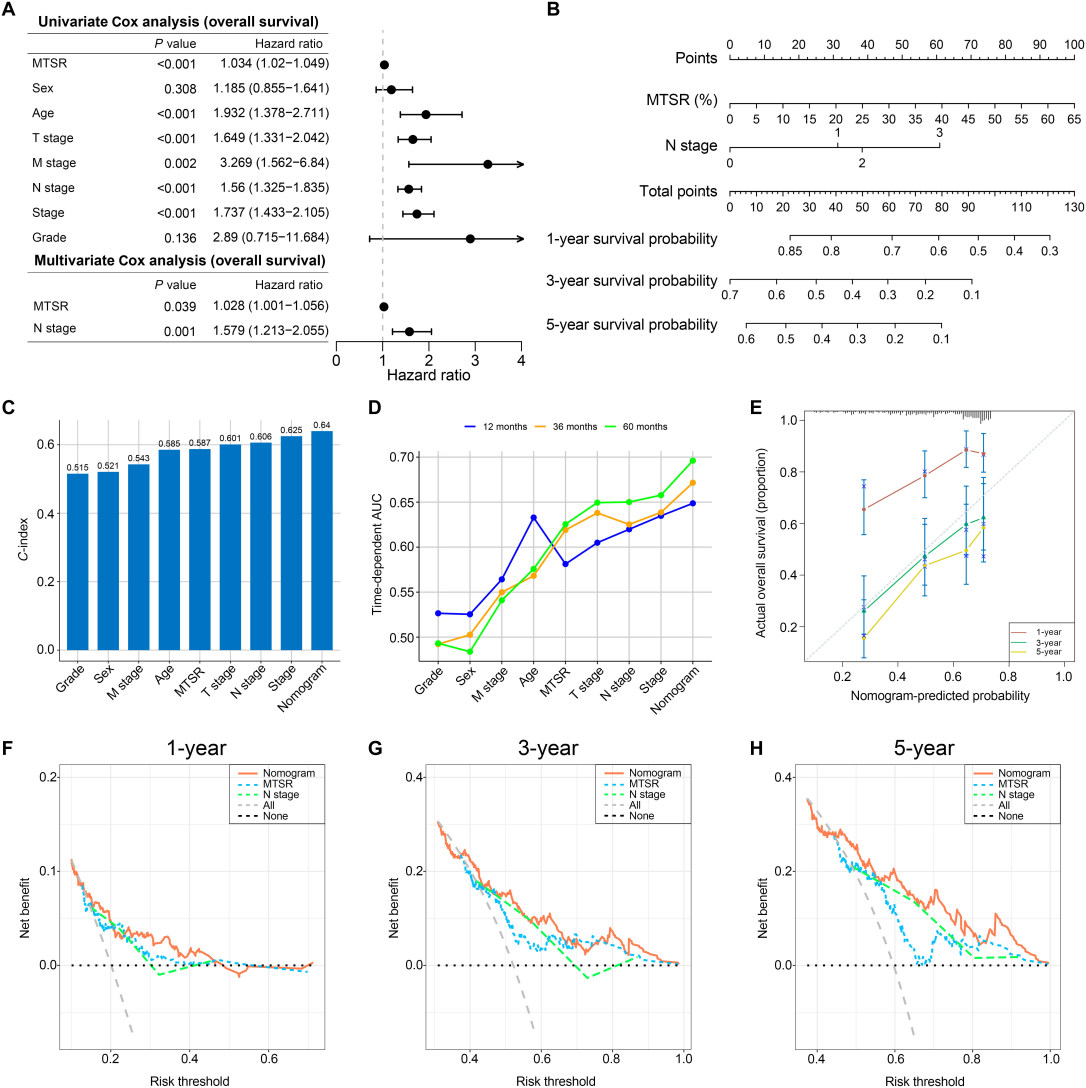
Development and validation of a prognostic nomogram incorporating MTSR and N stage. (A) Forest plot of univariate and multivariate Cox proportional hazards regression analysis in the TCGA bladder cancer cohort. (B) Nomogram predicting 1-, 3-, and 5-year OS probabilities in bladder cancer patients, based on MTSR and N stage. (C) Harrell’s concordance index (*C*-index) values for prognostic models based on MTSR, N stage, and the combined nomogram. (D) Time-dependent receiver operating characteristic (ROC) curves and area under the ROC curve (AUC) values for MTSR, N stage, and the nomogram at 1-, 3-, and 5-year time points. (E) Calibration plots for the nomogram at 1-, 3-, and 5-year survival, comparing predicted probabilities with observed outcomes. (F to H) Decision curve analysis (DCA) for MTSR, N stage, and the nomogram at 1-, 3-, and 5-year time points.

This study further utilized the TCGA BCa database to perform univariate and multivariate Cox analyses on the clinicopathological characteristics of BCa patients in relation to MTSR, aiming to construct a clinical prognostic model. The results indicated that MTSR and N stage were independent prognostic factors for BCa patients (Fig. 5A). Based on these 2 variables, a nomogram (Fig. 5B) was developed to predict the survival probabilities of BCa patients.

*C*-index analysis and time-independent area under the receiver operating characteristic (ROC) curve (AUC) analysis demonstrated that the predictive performance of MTSR was comparable to that of pathological features, suggesting that MTSR possesses an equivalent prognostic value (Fig. 5C and D). Moreover, the nomogram constructed with MTSR and N stage achieved the highest values in *C*-index and time-independent AUC analyses compared to other variables (Fig. 5C and D), highlighting its robust predictive performance for clinical prognosis.

Calibration curves revealed that the nomogram showed novel agreement between predicted and observed outcomes for 1-, 3-, and 5-year survival predictions (Fig. 5E). Decision curve analysis indicated that the nomogram outperformed MTSR and N stage alone in predicting survival probabilities at all time points, further demonstrating its clinical utility and superior prognostic value (Fig. 5F to H).

### Molecular characteristics of BCa with high vs. low MTSR

**Fig. 6.**
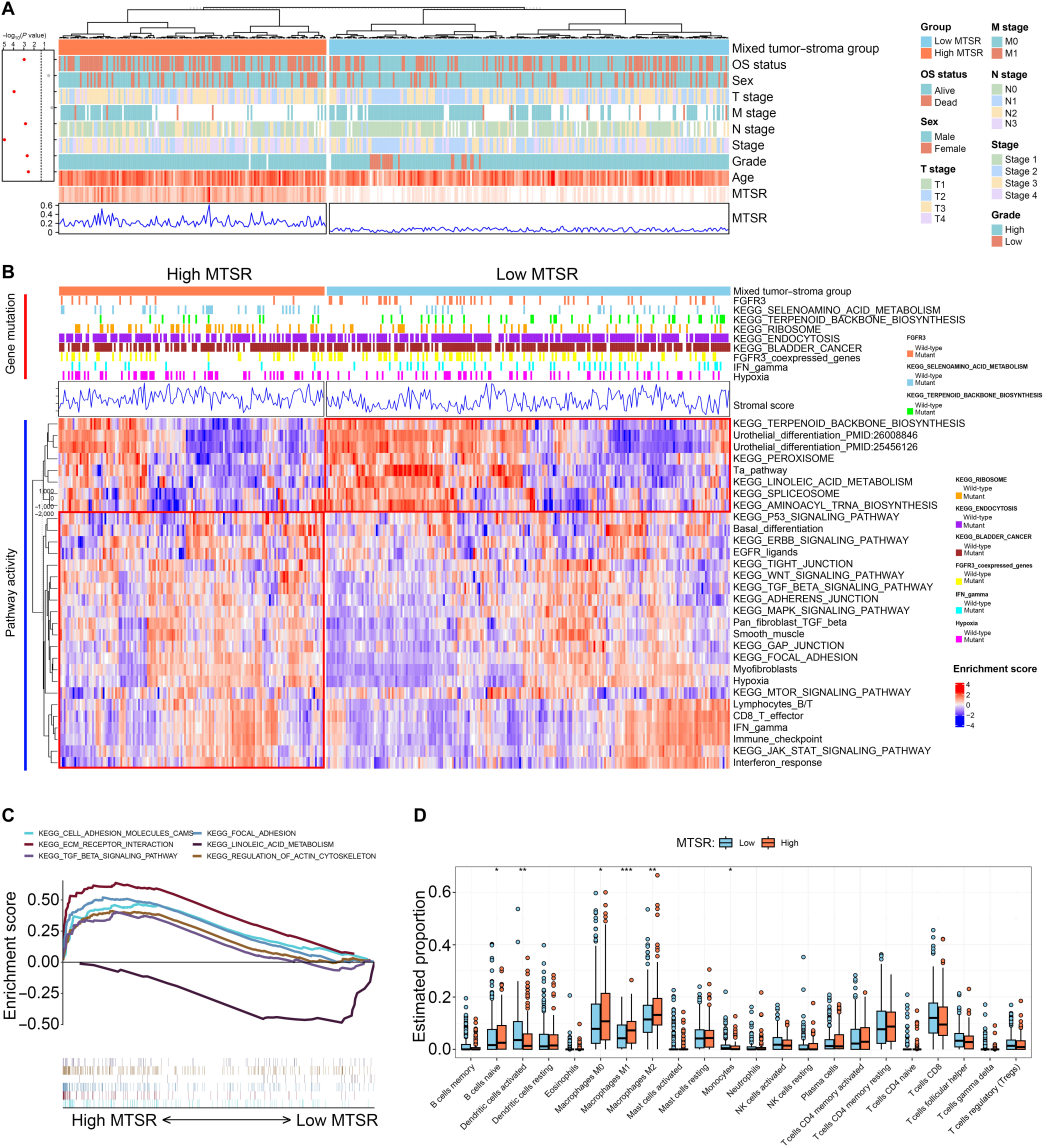
Molecular and immunological characteristics associated with high versus low MTSR in TCGA bladder cancer patients. (A) Heatmap showing the clinical characteristics of patients with high and low MTSRs. Variables include age, sex, tumor grade, and tumor–node–metastasis (TNM) stage. (B) Gene mutation and pathway activity analysis comparing high- and low-MTSR groups. (C) GSEA results comparing high- and low-MTSR groups. (D) Comparison of tumor-infiltrating immune cell (TIIC) proportions between high- and low-MTSR groups. NK cell, natural killer cell.

This study further characterized the clinical and molecular distinctions between BCa patients with high and low MTSR values in the TCGA bladder carcinoma (BLCA) cohort. Patients in the high-MTSR subgroup tended to be male and of older age and exhibited more advanced histological grade and TNM stage (Fig. 6A and Fig. S6). At the genomic level, low-MTSR tumors displayed a greater prevalence of mutations in FGFR3 and its co-expressed genes, along with marked enrichment in Kyoto Encyclopedia of Genes and Genomes (KEGG) pathways such as terpenoid backbone biosynthesis, endocytosis, BCa, and interferon-γ signaling. Conversely, high-MTSR tumors were characterized by higher mutation frequencies in pathways related to selenoamino acid metabolism, ribosome biogenesis, and hypoxia adaptation.

With respect to signaling activity, pathway enrichment profiling indicated that urothelial differentiation and the Ta pathway were preferentially activated in low-MTSR tumors, whereas basal differentiation and canonical oncogenic pathways—including WNT, pan-fibroblast transforming growth factor-β (TGF-β), and mTOR signaling—were predominant in the high-MTSR group (Fig. 6B). Gene set enrichment analysis (GSEA) further showed that linoleic acid metabolism was enriched in the low-MTSR group, while cell adhesion molecules, extracellular matrix (ECM)–receptor interaction, and TGF-β signaling were highly enriched in high-MTSR patients (Fig. 6C).

Regarding immune microenvironment characteristics, high-MTSR tumors exhibited increased infiltration of naive B cells and macrophage subsets (M0, M1, and M2), whereas low-MTSR tumors showed relatively higher proportions of activated dendritic cells and monocytes (Fig. 6D).

### Molecular characteristics of BCa with high vs. low MTSR at the single-cell level

In this study, we obtained 6 fresh BCa tissue samples from the Affiliated Guangdong Second Provincial General Hospital of Jinan University (GD2H) center for single-cell sequencing, with 3 samples in the high-MTSR group and 3 samples in the low-MTSR group. The cutoffs for high and low MTSRs were determined based on the median MTSR (4.275%) of all WSIs in the combined dataset. Detailed information regarding the H&E-stained WSIs, histological subtype distribution, and MTSR values for these single-cell sequencing BCa samples is provided in Fig. S7.

**Fig. 7.**
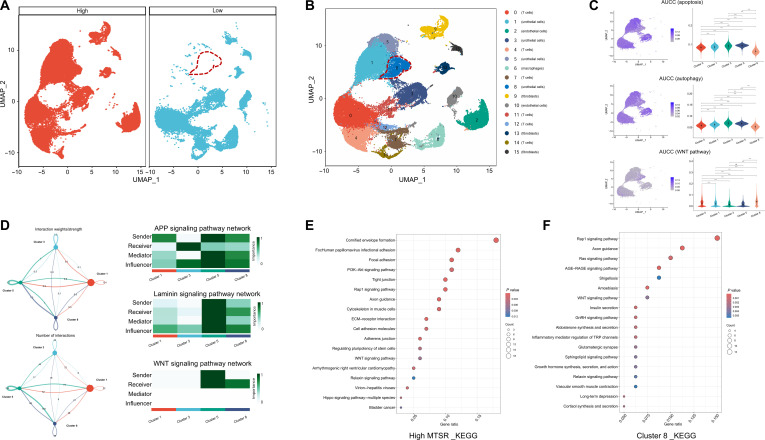
Single-cell transcriptomic analysis of bladder cancer samples with high versus low MTSR from the GD2H cohort (*n* = 6). (A) UMAP visualization of single-cell transcriptomes from 3 high-MTSR and 3 low-MTSR bladder cancer samples. (B) UMAP plot of unsupervised clustering identifying 16 major cell populations, including T cells, endothelial cells, urothelial cells, macrophages, and fibroblasts. Red dashed outlines in panels (A) and (B) indicate cell populations with marked differences between high- and low-MTSR samples. (C) Gene set activity scores (area under the ROC curve for cells [AUCC]) for apoptosis, autophagy, and WNT signaling pathways across cell clusters, displayed as UMAP heatmaps (left) and violin plots (right). (D) Cell–cell communication networks inferred from ligand–receptor interactions, with representative amyloid precursor protein (APP), laminin, and WNT signaling pathways. Left: interaction strength between clusters; right: heatmaps summarizing sender, receiver, mediator, and influencer roles. (E and F) KEGG pathway enrichment analysis comparing high- and low-MTSR groups as well as cluster 8 (urothelial cell enriched). Statistical significance is denoted as ****P < 0.0001. ns, not significant; PI3K/Akt, phosphoinositide 3-kinase/protein kinase B; ECM, extracellular matrix; AGE, advanced glycation end products; RAGE, receptor for advanced glycation end products; GnRH, gonadotropin-releasing hormone; TRP, transient receptor potential.

Key steps in the analysis of scRNA-seq data are shown in Fig. S8, revealing key RNA characteristics, including quality control metrics, relationships between RNA count and gene features, principal component analysis (PCA) of cellular variance, hierarchical clustering of sample relationships, and cell-type-specific gene expression patterns across various BCa cell types. Through scRNA-seq, distinct cell populations were observed in high-MTSR BCa samples compared with low-MTSR cases, highlighted by the red dashed outlines in Fig. 7A. Unsupervised uniform manifold approximation and projection (UMAP) clustering identified 16 major cell populations, including T cells, endothelial cells, urothelial cells, macrophages, and fibroblasts (Fig. 7B). Among these, cluster 8 was identified as a critical urothelial cell subtype. To assess functional states, we compared urothelial cell subtypes (clusters 1, 3, 5, and 8) with T cells (cluster 0) using area under the ROC curve for cells (AUCC) scoring (Fig. 7C). The results revealed that clusters 1, 3, and 5 exhibited higher apoptosis and autophagy activity than T cells, whereas cluster 8 showed reduced scores. Conversely, WNT signaling was markedly elevated in cluster 8 but attenuated in clusters 1, 3, and 5. Cell–cell communication analysis further illustrated the interaction networks among these urothelial cell subtypes, highlighting distinct roles as senders, receivers, and mediators within representative amyloid precursor protein, laminin, and WNT signaling pathways (Fig. 7D). KEGG pathway enrichment analysis of high-MTSR samples and cluster 8 (Fig. 7E and F) consistently demonstrated enrichment in tumor-progression- and epithelial–mesenchymal transition (EMT)-related pathways, including the phosphoinositide 3-kinase/protein kinase B (PI3K/Akt) signaling pathway, ECM–receptor interaction, Rap1 signaling, and WNT signaling.

### Gene module correlations and identification of key predictors for MTSR

**Fig. 8.**
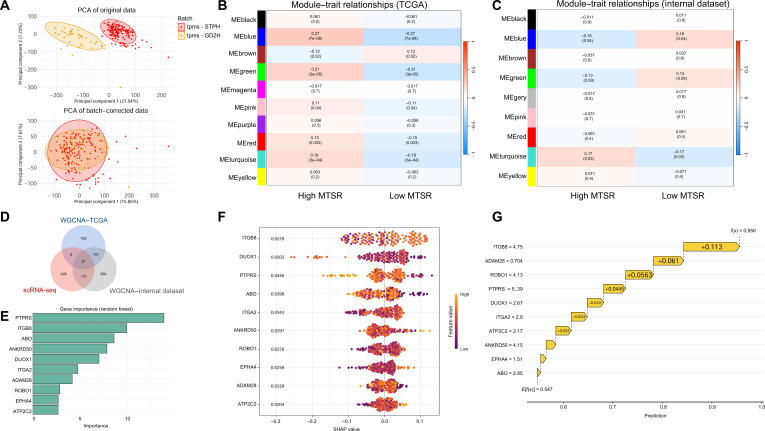
Workflow of integrated dataset analysis and machine learning for prognosis prediction in bladder cancer. (A) PCA of the original and batch-corrected datasets from GD2H and STPH, showing improved integration post-batch correction. (B) WGCNA module–trait relationships in the TCGA dataset for high- and low-MTSR groups. (C) WGCNA module–trait relationships in the integrated dataset for high- and low-MTSR groups, confirming consistency with the TCGA data. (D) Venn diagram showing the overlap between WGCNA-derived hub genes from the integrated/TCGA dataset and differentially expressed genes from cluster 8 (single-cell RNA sequencing [scRNA-seq]). (E) The top 10 gene importance scores generated by the random forest model pinpointed protein tyrosine phosphatase receptor sigma (PTPRS), ITGB8 integrin subunit beta 8 (ITGB8), and alpha 1-3-*N*-acetylgalactosaminyltransferase and alpha 1-3-galactosyltransferase (ABO) as the leading predictors. (F) The Shapley additive explanations (SHAP) bee swarm plot presents the impact magnitude and orientation that 10 genes exert on the MTSR prediction results. (G) The waterfall plot shows the positive class prediction values of 10 genes for the MTSR prediction results. tpms, transcripts per million.

As shown in Fig. 8A, the original datasets revealed clear batch differences between the GD2H and Shanghai Tenth People’s Hospital (STPH) cohorts, whereas after batch correction, the 2 groups largely overlapped in PCA space, indicating successful integration. In both the TCGA dataset and the integrated dataset, marked correlations were observed between MTSR status and multiple gene modules (Fig. 8B and C). Specifically, high-MTSR samples were positively correlated with several gene modules, while low-MTSR samples showed negative correlations, and these trends were consistent across both datasets. Next, we compared hub genes from the TCGA and integrated datasets with differentially expressed genes from cluster 8 identified by single-cell RNA sequences, and identified 21 overlapping candidate genes (Fig. 8D). Feature importance ranking of these 21 genes (Fig. 8E) revealed that protein tyrosine phosphatase receptor sigma (PTPRS), integrin subunit beta 8 (ITGB8), and alpha 1-3-*N*-acetylgalactosaminyltransferase and alpha 1-3-galactosyltransferase (ABO) ranked as the top predictors of MTSR status. Notably, ITGB8 not only showed high importance scores but also emerged as a key factor in Shapley additive explanations (SHAP) analysis. The SHAP bee swarm plot demonstrated that ITGB8 exerted the most prominent effect on the distribution and direction of predictions (Fig. 8F), while the SHAP waterfall plot further confirmed that ITGB8 contributed the strongest effect in driving model predictions toward high MTSR (Fig. 8G).

### Pseudotime analysis and regulatory networks of ITGB8-high urothelial cells

**Fig. 9.**
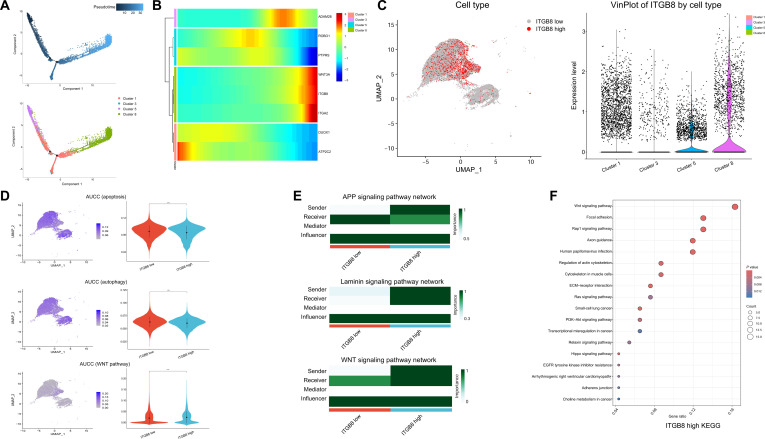
Analysis of urothelial cells and ITGB8 expression during pseudotime progression. (A) Pseudotime analysis reveals a progressive trajectory among urothelial cell clusters, with clusters 1, 3, 5, and 8 showing continuous development and cluster 8 representing the terminal stage. (B) The expression levels of ITGB8 and WNT family member 3A (WNT3A) rise markedly along pseudotime, with the highest expression observed in cluster 8. (C) Urothelial cells are classified into 2 categories based on ITGB8 expression: high and low, across clusters 1, 3, 5, and 8. (D) Gene expression scoring further highlights functional differences between ITGB8-high and ITGB8-low cells during urothelial progression. (E) Cell–cell communication analysis identifies interaction networks among different cell types in relation to ITGB8 expression. (F) KEGG pathway enrichment analysis shows that ITGB8-high cells are markedly involved in cancer-related signaling pathways.

We performed pseudotime analysis on urothelial cell subtypes (clusters 1, 3, 5, and 8) to infer their differentiation trajectories. The results showed that cluster 8 was positioned at the terminal stage of the trajectory, suggesting that it represents the final differentiated state of urothelial cells (Fig. 9A). A heatmap of gene expression further revealed a gradual increase in ITGB8 and WNT family member 3A (WNT3A) levels along pseudotime, with the highest expression observed in cluster 8 (Fig. 9B). Based on ITGB8 expression, urothelial cells were classified into high-expression and low-expression groups. ITGB8 was found to be predominantly enriched in cluster 8 (Fig. 9C). AUCC scoring demonstrated that compared with the low-expression group, ITGB8-high cells exhibited lower activity in apoptosis and autophagy pathways but higher activity in the WNT signaling pathway, a pattern consistent with the findings in Fig. 7C (Fig. 9D). Cell–cell communication analysis revealed that ITGB8-high cells were more likely to act as “senders” within signaling networks, exerting stronger regulatory influence on other cell populations (Fig. 9E). KEGG enrichment analysis further showed that the WNT signaling pathway was the most markedly enriched pathway in the ITGB8-high group, alongside several other cancer-related pathways (Fig. 9F).

We analyzed the major transcription factors enriched in ITGB8-high and ITGB8-low urothelial cells and observed distinct overall trends in their regulatory networks (Fig. S9A). In ITGB8-high cells, regulon specificity score ranking highlighted bromodomain PHD finger transcription factor (BPTF), SRY-box transcription factor 4, GATA binding protein 2 (GATA2)-extended, Kruppel-like factor 5 (KLF5), and POU class 3 homeobox 1 (POU3F1) as the top regulons (Fig. S9B). Single-cell UMAP visualizations further revealed the distribution of regulon activities across cell populations (Fig. S9C to G). BPTF exhibited strong activity in clusters 1, 5, and 8, with particularly high levels in cluster 8. Similarly, SOX4, GATA2-extended, KLF5, and POU3F1 showed preferential activity in ITGB8-high cells.

### Predicting MTSR by constructing an mpMRI-based radiomics model

**Fig. 10.**
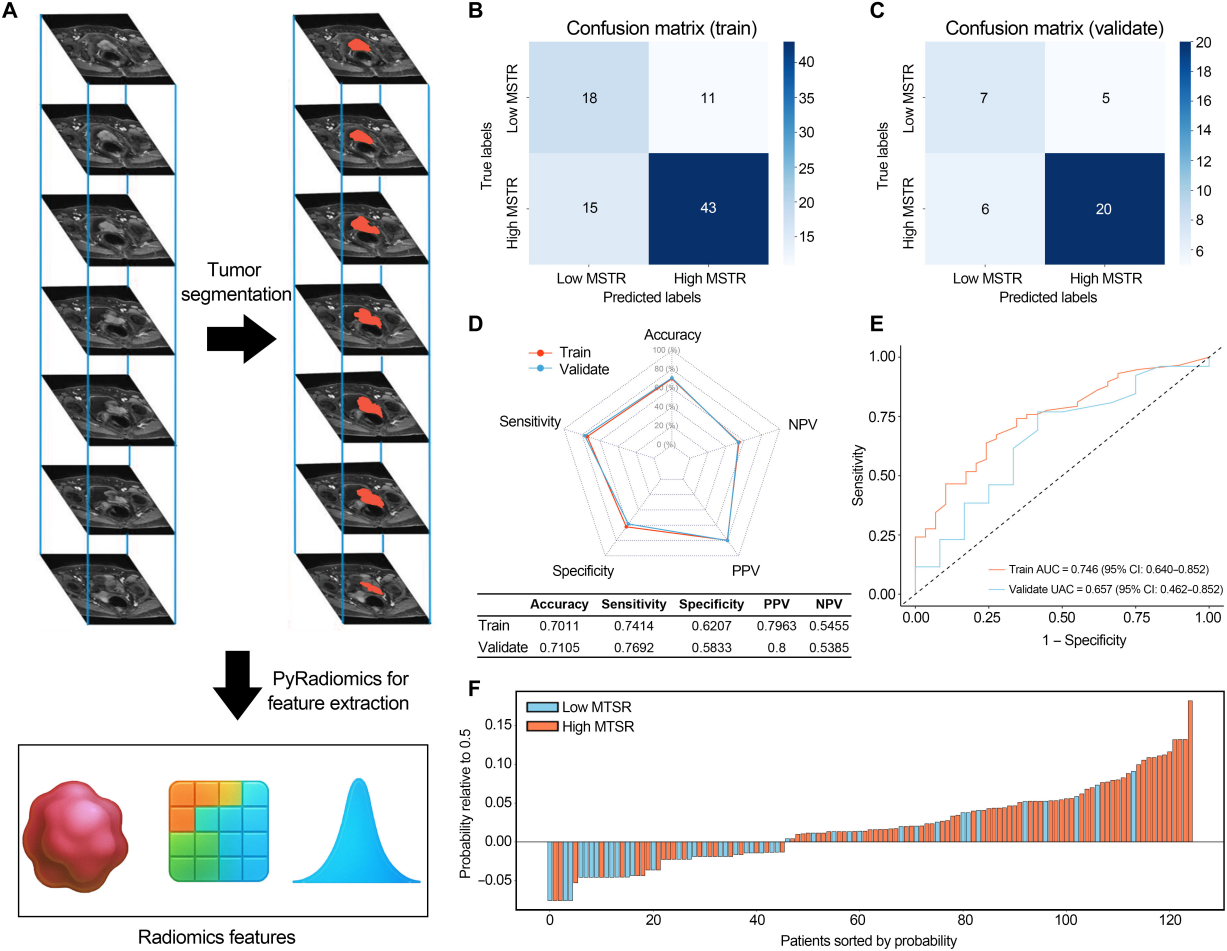
Results of the random forest model for predicting MTSR based on radiomics features. (A) Schematic of tumor ROI delineation and radiomics feature extraction. (B and C) Confusion matrices of the random forest model predictions on the training set (B) and validation set (C). (D) Radar charts showing the prediction results of the random forest model for MTSR in the training and validation sets. (E) ROC curves of the random forest model for MTSR prediction in the training and validation sets. (F) Waterfall plot of the random forest model predictions for MTSR. NPV, negative predictive value; PPV, positive predictive value.

In total, 1,781 quantitative radiomic descriptors were extracted from each patient’s mpMRI sequence using the PyRadiomics framework (Fig. 10A). Consequently, every patient contributed 3,562 imaging features in total, comprising 1,781 from the T2-weighted imaging sequence and 1,781 from the dynamic-contrast-enhanced sequence. The ICCs of these features ranged between 0.756 and 0.916, confirming good measurement consistency across repeated evaluations.

Within the training cohort, the 10 most informative radiomic features were identified through the minimum redundancy maximum relevance (mRMR) algorithm (Table S1) and used to develop a random forest classifier. Parameter optimization via grid search determined the best-performing model configuration: 13 decision trees, a maximum tree depth of 1, a minimum of 2 samples per node split, and at least 6 samples per leaf node.

Model predictive performance was visualized through a confusion matrix, indicating reliable MTSR classification in both training and validation cohorts (Fig. 10B). As illustrated in the radar chart, the random forest classifier achieved accuracies of 0.701 and 0.710 in the training and validation sets, respectively. The model maintained robust sensitivity (training: 0.741; validation: 0.769) and positive predictive value (training: 0.796; validation: 0.800) across datasets (Fig. 10C). ROC curve analysis yielded an AUC of 0.746 (95% CI: 0.640 to 0.852) for the training set and 0.657 (95% CI: 0.462 to 0.852) for the validation set, confirming the predictive strength of the model (Fig. 10D). Furthermore, the waterfall plot (Fig. 10E) graphically highlighted individual prediction outcomes, underscoring the consistency and stability of model performance.

## Discussion

BCa is a substantial clinical challenge due to its high progression and recurrence rates. Accurate pathological diagnosis is important for treatment decisions and survival prediction. Currently, the pathological diagnosis of BCa relies on manual slide review by pathologists. This process is time-consuming, subjective, and unable to accurately quantify the proportion of each tissue class within H&E-stained slides. In addition, pathologists may also introduce interobserver variability, particularly in complex cases where tissue types are ambiguous or the margins between tumor and normal tissue are not well defined.

To address these challenges, we developed a deep learning model for the automated analysis of H&E-stained WSIs in BCa. In comparison to conventional pathological assessments, our model provides a more objective and reproducible method of classifying diverse tissue types [27,28]. The model showed consistently high accuracy in identifying multiple tissue types, achieving over 90% accuracy on both internal and external validation datasets with Cohen’s kappa values exceeding 0.95. The model’s ability to identify tumor tissues with high precision is particularly valuable for discovering subtle tumor lesions that might be missed by pathologists. In addition, while pathologists remain central to cancer diagnosis, integrating AI models could help in reducing interobserver variability and improving diagnostic consistency, especially in high-volume settings and complex cases.

Our approach introduces the MTSR, which utilizes deep learning for automated, patch-level segmentation of WSIs. Unlike previous manual assessments [29,30], our method classifies tissue into 10 categories, including tumor and mixed tumor–stroma regions, enhancing precision and reducing interobserver variability. By leveraging a large training dataset from the TCGA BCa database and external validation, our MTSR offers a more accurate, reproducible, and scalable solution for quantifying tumor–stroma interactions, with greater potential for clinical application. Our analysis further reveals that BCa patients with a higher MTSR exhibit a poorer prognosis, consistent with findings in other cancers [31–34]. In this study, MTSR was the only useful prognostic marker for BCa among the 9 tissue classes. BCa patients with a higher MTSR had worse OS across both the TCGA dataset and 5 external validation cohorts. This result revealed that the MTSR, particularly in the invasive areas of the tumor, may serve as a novel determinant of tumor recurrence and progression [29]. The consistency of MTSR’s prognostic value across 5 external datasets further supports its potential as a reliable marker for prognosis prediction. Crucially, MTSR remained an independent predictor of outcome after adjustment for conventional factors such as TNM stage, underscoring its potential to augment existing prognostic models.

The prognostic significance of MTSR can be explained through bioinformatics. The MTSR reflects the degree of myofibroblast-driven stromal activation that underpins several hallmarks of malignancy, such as immune evasion [35], angiogenesis [29], and ECM remodeling [36]. A high MTSR is associated with a more aggressive tumor microenvironment, where tumor–stroma interactions could motivate tumor invasion and metastasis. Our integrative bioinformatics comparison of high- versus low-MTSR groups further revealed divergent molecular programs. Notably, BCa patients in the high-MTSR group exhibited higher mutation frequencies in pathways related to hypoxia and ribosome biogenesis, while BCa patients in the low-MTSR group showed enrichment in pathways related to urothelial differentiation. These findings reveal the distinct biological processes that drive tumor progression between low and high MTSRs and suggest potential treatment targets for therapeutic intervention [29,36,37]. For example, patients with a high MTSR may benefit from more aggressive treatment options, including neoadjuvant chemotherapy [29,38], while those with a lower MTSR may be candidates for less intensive regimens.

The scRNA-seq analysis provided a high-resolution view of the cellular composition in BCa tumors with varying MTSR levels. We observed distinct cellular subtypes and functional states between high- and low-MTSR tumors, particularly within the urothelial cell population. Pseudotime analysis shows that urothelial cells with high ITGB8 expression (mainly concentrated in cluster 8) are located at the terminal end of the differentiation trajectory, suggesting that these cells may be in a terminally differentiated state but still exhibit invasive characteristics. These features are likely mediated by the WNT signaling pathway and ITGB8. Research has shown that ITGB8 activates TGF-β through its cytoplasmic domain, which in turn drives the WNT/β-catenin pathway in brain endothelial cells to regulate neurovascular development [39]. Gene expression patterns further support the hypothesis that high-MTSR tumors exhibit features of tumor progression, including reduced apoptosis and enhanced WNT signaling, consistent with the concept of EMT. Existing evidence shows that ITGB8, as the integrin β8 subunit, forms a heterodimer with the αv subunit, binds to latent TGF-β in the ECM, and induces its activation, leading to the release of active TGF-β1/β3. This paracrine activation stimulates stromal fibroblasts and immune cells, driving collagen deposition, EMT, and immune suppression [40]. In lung cancer models, high expression of ITGB8 in cancer cells can induce cancer-associated fibroblasts (CAFs) to contract collagen, polarize macrophages to the M2 phenotype, and upregulate ITGB8 in tumor cells, thus forming a positive feedback loop [41]. Future work should exploit BCa/CAF or macrophage co-cultures, ITGB8-blocking antibodies, and orthotopic mouse models to test its pro-fibrotic and immunosuppressive roles and to examine the synergy between TGF-β and PD-1 blockade. This emphasizes the dynamic nature of tumor evolution, where the stromal environment contributes to driving tumor cell differentiation and progression [42–44].

The identification of transcription factors such as BPTF, SOX4, and KLF5 in ITGB8-high cells further implicates these factors in regulating tumor–stroma interactions and mediating the aggressive phenotype associated with a high MTSR. BPTF has been shown to contribute to erlotinib resistance in gastric cancer by regulating the c-MYC/PLCG1/p-Erk axis [45]. SOX4 promotes cell proliferation and EMT by activating the TGF-β/Smad2/3 pathway in inflammatory environments [46]. KLF5 is involved in muscle atrophy and may regulate this process through the WNT/β-catenin pathway [47]. These factors are crucial in tumor progression and may influence therapeutic responses. The ability to trace the regulatory networks of these transcription factors at the single-cell level offers novel insights into the molecular mechanisms driving BCa progression, providing potential targets for therapeutic intervention.

The ability to predict MTSR preoperatively offers meaningful advantages in clinical practice, as it allows for early risk stratification and treatment planning. A radiomics model based on preoperative mpMRI scans has shown modest results in predicting MTSR, demonstrating the feasibility of using noninvasive imaging techniques to predict the prognostic significance of MTSR in BCa patients. MTSR reflects the proportion of tumor mixed with stroma and thereby the ECM density, fibroblast activity, and immune cell admixture. Texture features derived from mpMRI often capture microstructural heterogeneity related to collagen deposition, edema, and perfusion restrictions; these correlate with malignant grade and variant histology in bladder MRI studies, lending plausibility that macroscale imaging can encode microenvironmental composition [24,25,48].

Despite the promising results, this study has certain limitations. Firstly, while the external validation datasets are large, they are still limited in diversity, and the model’s development based on a small, single-center sample raises concerns about its generalizability. Future studies with larger, more diverse patient cohorts are essential to validate the generalizability of MTSR. Secondly, although our study established the prognostic value of MTSR, the underlying mechanisms driving stromal-tumor interactions require further investigation. Additionally, the MRI radiomics model demonstrates modest accuracy (AUC 0.71), and its clinical utility should be interpreted with caution. Future research focusing on the molecular and cellular dynamics of the tumor microenvironment, particularly in the context of MTSR, will enhance our understanding of its role in BCa progression. Lastly, exploring the potential of integrating MTSR with other biomarkers or treatment response indicators could lead to more comprehensive prognostic models, improving patient management.

In conclusion, this study highlights the prognostic significance of MTSR in BCa and provides compelling evidence for the potential of deep learning and multi-omics approaches in improving patient stratification and survival prediction. These findings lay the groundwork for further research into therapeutic strategies targeting the tumor microenvironment and offer a pathway toward more personalized and effective treatment of BCa patients.

## Methods

### Patients

The study protocol received approval from the ethics committees of all participating centers, including GD2H (Approval No. 2024-KY-KZ-128-01), STPH (Approval No. 24KT68), the Third Affiliated Hospital of Sun Yat-Sen University (ZSSY; Approval No. 2022-02-299-02), People’s Hospital of Puning City (PN; Approval No. 2024-KY-59), and the First Affiliated Hospital of Wannan Medical College (Yijishan Hospital) (YJSH; Approval No. 2024-72). Written informed consent was obtained from every participant prior to inclusion.

This retrospective study comprised the TCGA BCa dataset and 5 external validation datasets (as mentioned above), employed for developing and validating a CNN model to identify various histological subtypes of H&E-stained WSIs in BCa. The prognostic significance of CNN-derived histological subtype proportions was also assessed using follow-up data from these datasets. For the external validation datasets, BCa patients who underwent curative-intent surgery and had available paraffin-embedded tumor samples were enrolled between April 2014 and May 2024. The following exclusion criteria were applied across all cohorts: (a) lack of tumor tissue block on the slide; (b) insufficient scan quality or insufficient stain quality; (c) lack of clinical information or postoperative pathological information; and (d) patients who had received chemotherapy, radiotherapy, or Bacillus Calmette–Guérin treatment within 30 d before surgery were excluded. The primary endpoint was OS, defined as the period from the date of surgery to death from any cause. Survival follow-up was conducted until the most recent clinical visit, at which time each participant’s vital status was recorded.

### Procedures

An overview of the study workflow is provided in Fig. 1. For subsequent analyses, standard H&E-stained slides representing the most infiltrative tumor areas were identified by an experienced pathologist (B.Z., >10 years in BCa pathology). Slide selection was performed independently and blinded to clinical data or patient outcomes. The chosen slides were then scanned into WSIs at ×40 magnification using a KF-PRO-120/005 digital scanner (KFBIO, China). Image patches in WSIs were classified into 10 classes: background and 9 tissue classes (namely, adipose, burn or deformation, inflammatory, mixed tumor–stroma, muscle, necrosis, stroma, tumor, and vessel). QuPath (version 0.3.2) was utilized for image annotation across these 10 classes. Two junior pathologists (Y.Y.H. and J.F.), each with over 5 years of experience in BCa pathology, independently identified and manually delineated regions of interest (ROIs) for each tissue class. A senior pathologist (B.Z.) reviewed and validated all annotations, resolving discrepancies through consensus. Patches were defined as “tumor” if the tumor occupied more than 90% of the patch area and as “mixed tumor–stroma patch” if the tumor occupied less than 90% of the patch area and coexisted with stroma. Image patches sized 224 × 224 pixels (at ×20 magnification) were then extracted from the annotated regions. To ensure consistency in color representation across different tissue sections, color correction was performed using Reinhard color normalization [49].

### Training and assessment of the neural network

The TCGA BCa database was selected for training and internal validation due to its extensive WSIs, comprehensive clinical data, and detailed molecular information. To rigorously prevent information leakage, the division between the training and internal validation sets was strictly performed at the patient level. This ensured that all WSIs from any single patient were exclusively assigned to either the training set or the internal validation set, guaranteeing the complete independence of the validation set. Specifically, WSIs from a randomly selected subset of patients (comprising 2,231,536 image patches, including “background” patches, from 80 WSIs) formed the training set. WSIs from a separate, nonoverlapping group of patients (comprising 530,423 image patches, including “background” patches, from 20 WSIs) constituted the internal validation set. External validation was subsequently performed using an independent dataset from 5 clinical centers. The terminal classification layer of a pre-trained ResNet50 CNN was modified to output 10 categories, aligning with the defined tissue classes. Model optimization was achieved through transfer learning using stochastic gradient descent, enabling the discrimination of background regions and 9 histological tissue types in BCa WSIs. Training was performed on a workstation equipped with an NVIDIA RTX 4090 graphics processing unit (GPU), with a mini-batch size of 160 and an initial learning rate of 0.01. To promote stable model convergence, a per-batch exponential decay rate of 0.9999 was applied to the learning rate, and training proceeded for 50 epochs. Model accuracy and Cohen’s *κ* coefficient were calculated to assess performance on independent external datasets. Deep feature representations from the final average pooling layer were visualized through t-SNE to evaluate class separation.

After training, the CNN was applied for patch-level segmentation of WSIs. Image tiles of 224 × 224 pixels were extracted from H&E-stained slides scanned at ×20 magnification using a nonoverlapping sliding window strategy. Edge detection preprocessing was then conducted to remove patches with insufficient tissue regions. Grayscale conversion followed by the Canny operator was used to determine the edge density, and tiles with less than 2% edge pixels were discarded (OpenCV v4.10). The remaining patches were standardized for color distribution via Reinhard normalization to minimize interslide variation. These normalized tiles were subsequently input into the CNN to obtain probabilistic predictions, with each patch assigned to the class showing the highest confidence score. All segmentation and inference procedures were GPU-accelerated for computational efficiency. For visualization, distinct colors were used to represent the 9 different tissue classes (Fig. 1). Finally, the tissue class ratio was calculated using the following formula:

$$\begin{array}{c} \begin{array}{c} \begin{array}{c} \text{Tissue class ratio}\;\left(\%\right)=\frac{\text{Area of}\;a\;\text{specific histological type}}{\text{Total area of}\;9\;\text{histological types}}\times 100\% \end{array} \end{array} \end{array}$$ (1)

### Evaluation of the prognostic significance of each tissue class ratio

Initially, individual univariate Cox proportional hazards analyses were performed to explore associations between each of the 9 tissue type ratios and OS. Continuous variables were dichotomized using optimal cutoff values identified via maximally selected rank statistics (survminer v0.4.8). The estimated HRs with corresponding 95% CIs from each dataset were then combined through a 2-stage meta-analytic approach implemented with the meta (v6.2) and metafor (v4.2) R packages. Study heterogeneity was evaluated using the *I*^2^ index and Cochran’s *Q* statistic; datasets meeting the criteria of *I*^2^ < 25% and *Q* test *P* > 0.05 were synthesized under a fixed-effects inverse-variance model, while those showing marked heterogeneity were analyzed using the DerSimonian–Laird random-effects framework. Funnel-plot symmetry and Egger’s regression were applied to assess potential small-sample bias.

Kaplan–Meier survival curves were plotted based on the optimized thresholds, and group differences were tested by the log-rank method. Within the TCGA cohort, predictors significant in the univariate step were further incorporated into a multivariate Cox regression to develop a composite prognostic index, which was subsequently visualized as a nomogram. The proportional hazards assumption was verified via Schoenfeld residual testing, and internal robustness was assessed through bootstrap resampling (1,000 iterations). The prognostic discrimination and clinical applicability of the model were evaluated using Harrell’s concordance index (*C*-index), time-dependent ROC curves with integrated AUC, calibration assessments, and decision curve analysis.

### Bioinformatics analysis

RNA-seq expression profiles and somatic mutation data for BCa were obtained from TCGA through the Genomic Data Commons interface. Raw count data were normalized to fragments per kilobase of transcript per million mapped reads (FPKM) to account for transcript length and sequencing depth, followed by log_2_(FPKM + 1) transformation. Genes exhibiting FPKM values below 1 in more than half of the cases were excluded to eliminate transcripts with extremely low expression levels.

Pathway enrichment was explored through GSEA (v4.1.0) using curated gene sets, while gene set variation analysis provided sample-specific enrichment scores to quantify pathway activities. Complementary enrichment testing was conducted with the clusterProfiler R package (v4.4.0), employing pathway annotations derived from the KEGG database (2021 release).

To infer immune cell composition, CIBERSORTx analysis was carried out using the LM22 gene signature and 1,000 permutations to estimate the relative fractions of 22 leukocyte populations. In addition, immune and stromal infiltration levels were computed via the ESTIMATE algorithm (estimate R package, v1.0.13), generating immune, stromal, and composite ESTIMATE indices that reflect the nonmalignant cell content within each tumor sample.

### Single-cell RNA sequence analysis

From the GD2H cohort, 3 BCa samples representing low- and high-mixed MTSR groups were selected for scRNA-seq (sequencing details available under Genome Sequence Archive for Human [GSA-Human] accession no. HRA009938). Data preprocessing was performed in Seurat (v4.0.3), encompassing quality control, normalization, and scaling steps. Quality control thresholds were applied as follows: minimum RNA molecule count of 1,000, maximum RNA molecule count of 50,000, minimum detected genes of 200, maximum detected genes of 6,000, maximum mitochondrial gene percentage of 15%, and maximum ribosomal gene percentage of 100%. The number of highly variable genes selected was 2,000. Dimensionality reduction was achieved through PCA, and potential batch effects were adjusted using the Harmony algorithm (v0.1.0) [50]. Cells were clustered within the Seurat pipeline based on the shared-nearest-neighbor modularity optimization method (FindClusters, resolution = 0.2). The 2-dimensional representation of cellular subpopulations was visualized using UMAP to facilitate interpretation of intratumoral heterogeneity.

Cell populations were annotated using canonical lineage markers [51]. Differentially expressed genes between specific clusters or experimental conditions were identified via the FindMarkers function in Seurat. For differential analysis, the significance threshold for *P* values was set to *P*_adj_ < 0.05, and Benjamini–Hochberg false discovery rate correction was applied to adjust for multiple testing. Biological function and pathway enrichment analyses were subsequently performed using clusterProfiler (v4.2.2) in conjunction with org.Hs.eg.db (v3.13.0), emphasizing processes related to EMT and associated oncogenic pathways such as PI3K and WNT signaling. T cells, especially CD8+ effector T cells, play a key role in immune surveillance by killing tumor cells, making them a good reference for normal immune function [52] Using T cells as a reference group helps quantify the tumor’s “immune deviation”, such as apoptosis resistance and metabolic reprogramming. AUCC scoring [53,54] compares programmed cell death and related pathway activity scores between clusters to understand the unique functional states of subpopulations.

To delineate dynamic transcriptional changes, pseudotime trajectory [55] reconstruction was conducted using the Monocle R package (v2.26.0), enabling visualization of urothelial cell state transitions during tumor evolution. Regulatory network inference was carried out with the Single-Cell Regulatory Network Inference and Clustering (SCENIC) framework [56] to identify transcription factor–target modules (regulons) and quantify their activities across cellular states. This integrative approach revealed key transcriptional regulators underlying phenotypic diversity within tumor cell populations.

### Weighted gene co-expression network analysis

RNA-seq data from BCa samples in the GD2H and STPH cohorts were obtained for integrative analysis (sequencing procedures are described under GSA-Human accession no. HRA009938). These datasets were merged to create an internal combined cohort for subsequent computational analyses. PCA was used to evaluate dataset consistency before and after batch adjustment. Application of the Harmony algorithm (v0.1.0) effectively minimized technical biases, resulting in enhanced cross-cohort comparability and improved sample alignment. The Harmony integration was performed using the default parameters (theta = 2, lambda = 1) on the top 50 principal components.

Within the TCGA BCa dataset, cases were stratified into high- and low-MTSR categories according to the model’s predicted scores. Weighted gene co-expression network analysis (WGCNA) was independently performed for both the TCGA and internal datasets to identify gene modules correlated with MTSR classification. Differential analysis of WGCNA modules was performed using an adjusted *P*-value threshold of *P*_adj_ < 0.05. Multiple testing correction was conducted using Benjamini–Hochberg false discovery rate correction. Overlapping hub genes were then determined by intersecting WGCNA module genes with the differentially expressed genes from cluster 8 identified in the scRNA-seq analysis, visualized using a Venn diagram.

To further pinpoint the essential molecular determinants of MTSR, a random forest algorithm was applied to the internal dataset. Gene importance scores were derived to rank features according to their predictive contribution, and the 10 most informative genes were retained for downstream analysis. Model interpretability was examined using SHAP, which quantified the relative influence of each gene on the model’s output and predictive behavior.

### Radiomic data acquisition and model construction

In this work, a radiomics-based predictive model was constructed to estimate MTSR using preoperative mpMRI scans from BCa patients in the STPH cohort. Eligible cases met the following inclusion criteria: (a) histologically confirmed urothelial carcinoma of the bladder, (b) mpMRI examination performed within 20 d before surgery, and (c) availability of corresponding H&E-stained WSIs. Exclusion criteria included the following: (a) prior exposure to chemotherapy, radiotherapy, transurethral resection of bladder tumor, or Bacillus Calmette–Guérin treatment before mpMRI acquisition; (b) suboptimal mpMRI image quality; and (c) unclear or non-delineable tumor margins. Ultimately, 125 H&E-stained WSIs from 109 individuals with matched mpMRI data were incorporated into the radiomics workflow. These cases were randomly assigned to the training and validation cohorts at a 7:3 ratio.

For image analysis, mpMRI data in DICOM format were processed for radiomic feature extraction. Tumor ROIs were manually outlined using the ITK-SNAP software, and quantitative features were extracted via the PyRadiomics package (http://PyRadiomics.readthedocs.io/en [57]). Features were extracted from T2-weighted imaging and dynamic-contrast-enhanced sequences, as these sequences provide complementary information on anatomical and vascular properties, respectively. The diffusion-weighted imaging sequence was excluded from the analysis due to inconsistencies in *b* values across the dataset, which could have impacted the reproducibility of the results. In cases with multiple BCa lesions, the largest lesion was selected for tumor boundary delineation and radiomic feature extraction. Feature reproducibility was assessed using the ICC. Comprehensive procedures for tumor contouring, feature extraction, ICC computation, and *z*-score normalization have been detailed previously [58].

Following preprocessing, the mRMR method was applied to the training dataset to identify the 10 most informative radiomic descriptors. These selected features were subsequently used to build a random forest classifier for MTSR categorization (high vs. low). To optimize model performance, a grid search strategy was employed to tune hyperparameters. Specifically, the number of trees ranged from 5 to 150, the maximum tree depth from 1 to 10, minimum samples required for node splitting from 2 to 10, and minimum samples per leaf from 1 to 6.

The final optimized random forest model was evaluated in both the training and validation cohorts using multiple performance indicators, including AUC, accuracy, sensitivity, specificity, positive predictive value, and negative predictive value.

### Statistical analysis

Continuous variables following a normal distribution were analyzed using 2-tailed Student *t* tests, while nonnormally distributed data were assessed with the Mann–Whitney *U* test. Differences in categorical variables—including age, sex, histological grade, TNM stage, and gene mutation status—between the 2 groups were examined using the chi-square test. All statistical analyses were performed in R software (v4.0.3). Deep learning model development and evaluation were implemented in Python (v3.12) within the PyTorch framework (v2.4).

## Ethical Approval

This study was approved by the Medical Ethics Committees of GD2H (Approval No. 2024-KY-KZ-128-01), STPH (Approval No. 24KT68), ZSSY (Approval No. 2022-02-299-02), PN (Approval No. 2024-KY-59), and YJSH (Approval No, 2024-72). Written informed consent was obtained from all participants involved in the study.

## Data Availability

The datasets generated and/or analyzed during the current study are available in the Genome Sequence Archive (*Genomics, Proteomics & Bioinformatics*, 2021) in the National Genomics Data Center (*Nucleic Acids Research*, 2022) and the China National Center for Bioinformation/Beijing Institute of Genomics, Chinese Academy of Sciences (HRA009938, https://ngdc.cncb.ac.cn/gsa-human). The TCGA whole-slide data are available from the National Institutes of Health Genomic Data Commons (https://portal.gdc.cancer.gov). To protect patient privacy, pathology image datasets and other patient-related data of 5 external validation datasets are not publicly accessible, but all data are available upon reasonable request emailed to the corresponding authors. To gain access, data requestors will need to sign a data-access agreement.
