# Supplementary material for: A Deep Learning-Generated Mixed Tumor–Stroma Ratio for Prognostic Stratification and Multi-omics Profiling in Bladder Cancer
Source: Research (Wash D C). 2026 Jan 26;9:1053. doi: 10.34133/research.1053 (PMC12833823; doi:10.34133/research.1053)
Supplement: Supplementary 1 — Figs. S1 to S9 Table S1 [file research.1053.f1.docx]

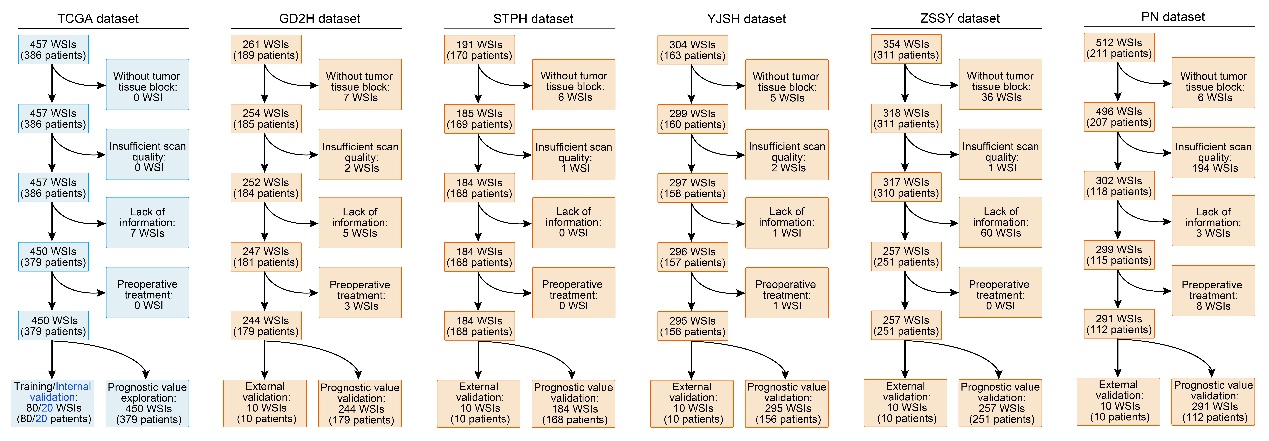


**Supplementary Figure 1** Flowchart of patient selection for each dataset.


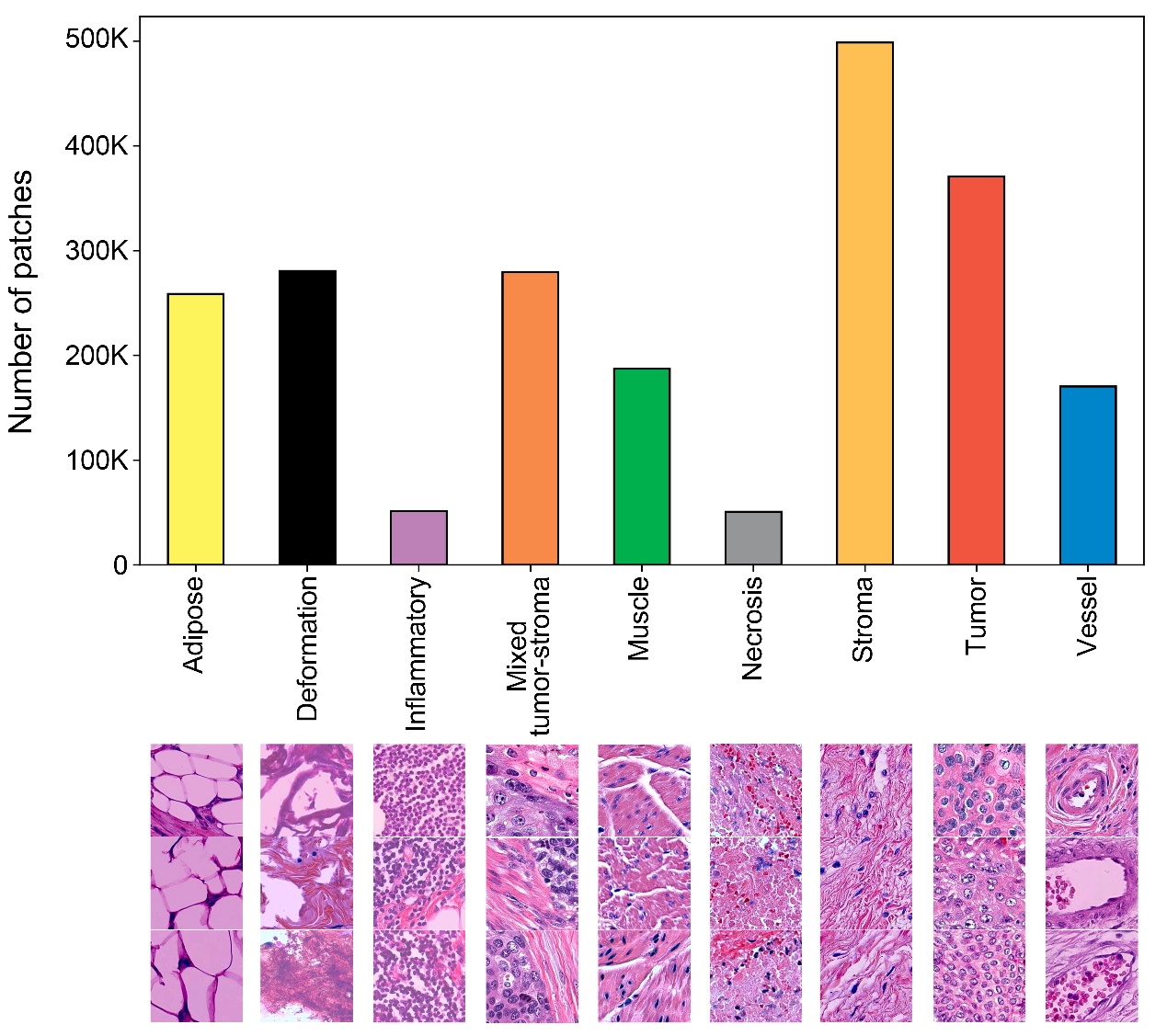


**Supplementary Figure 2** The number and representative images of each histological type in the TCGA training dataset.


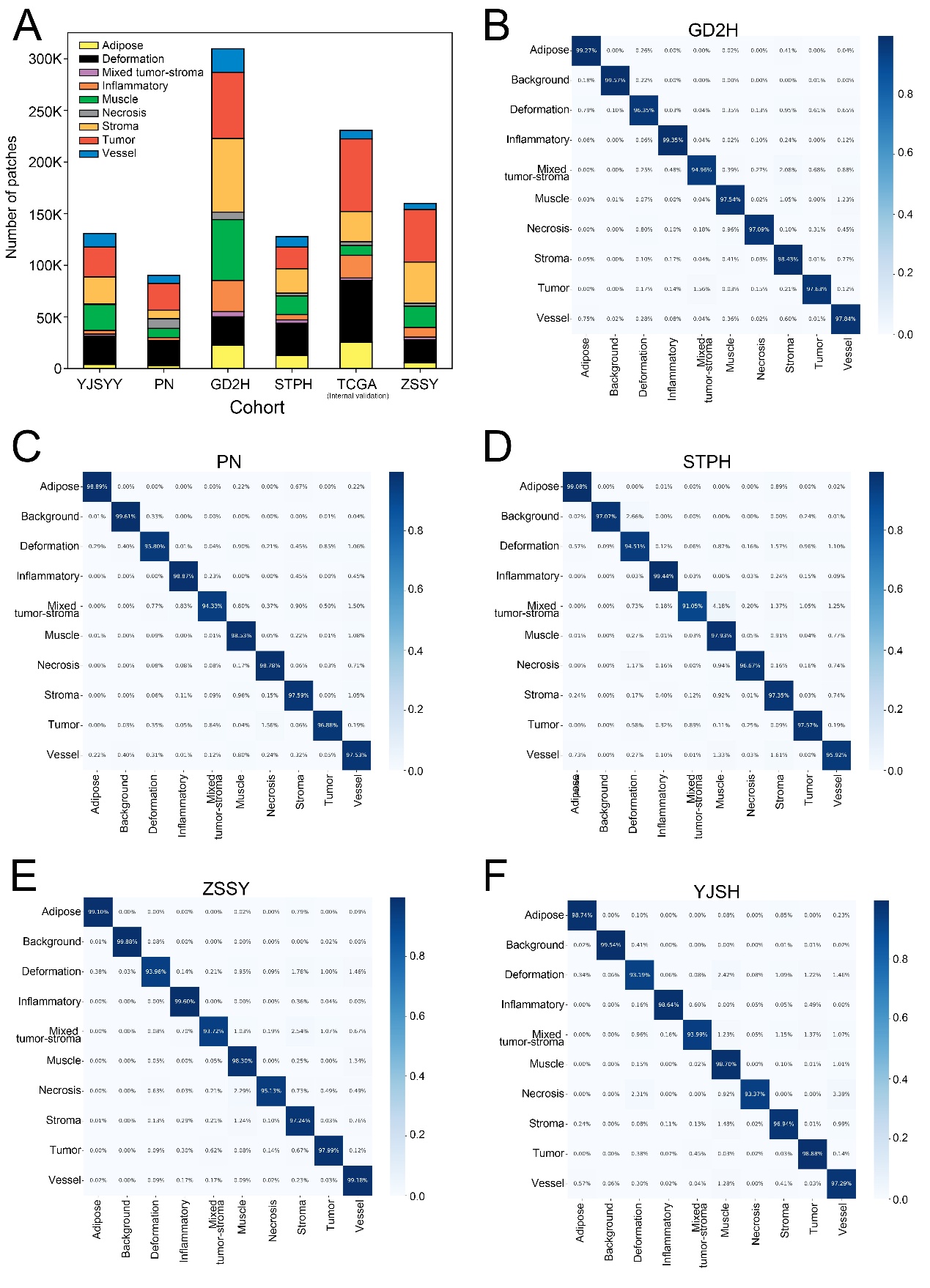


**Supplementary Figure 3** Trained ResNet50 model performance in external validation datasets. (A) Number of patches for each tissue class in the external validation datasets (GD2H, STPH, ZSSY, PN, YJSH). (B–F) Confusion matrices showing the classification accuracy of the trained ResNet50 model for each tissue class in GD2H (B), PN (C), STPH (D), ZSSY (E) and YJSH (F). Diagonal elements represent the percentage of correctly classified patches.


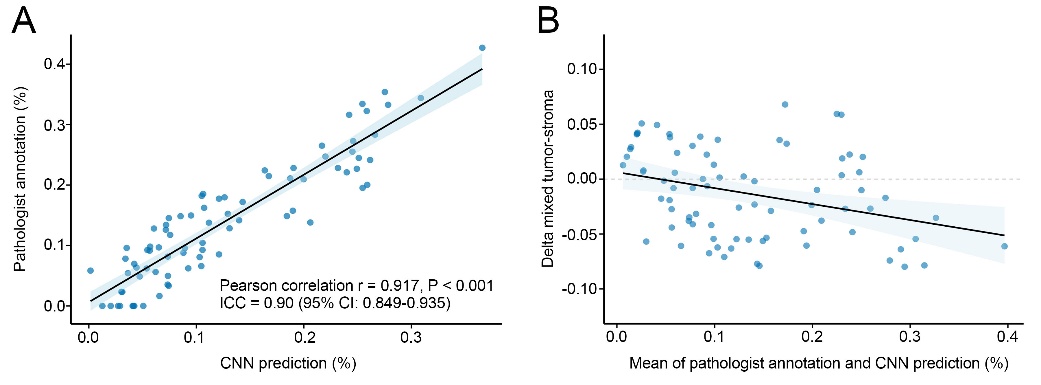


**Supplementary Figure 4** Validation of the CNN-predicted MTSR and agreement with pathologist annotations. (A) Scatter plot illustrating the correlation between the MTSR values predicted by the CNN and those annotated by the pathologist in the TCGA training dataset. (B) Bland–Altman plot assessing the agreement between the CNN-predicted and pathologist-annotated MTSR values. The mean difference is shown as a solid line, and the limits of agreement (±1.96 SD) are indicated by dashed lines.


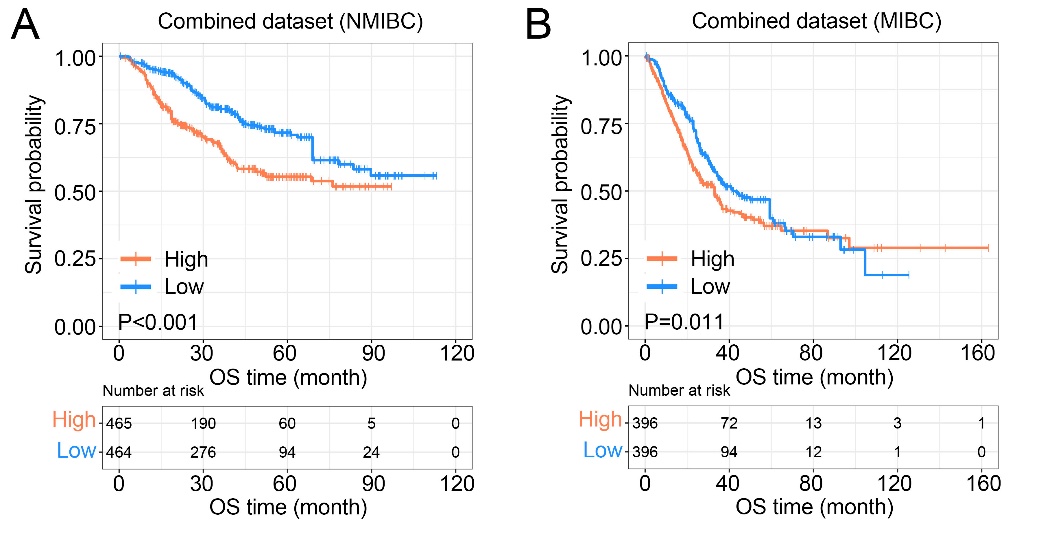


**Supplementary Figure 5** Kaplan-Meier survival analysis of OS in BCa patients stratified by MTSR in the NMIBC and MIBC subgroups from the combined dataset. (A) Kaplan–Meier survival curve for OS in the NMIBC subgroup. (B) Kaplan–Meier survival curve for OS in the MIBC subgroup.


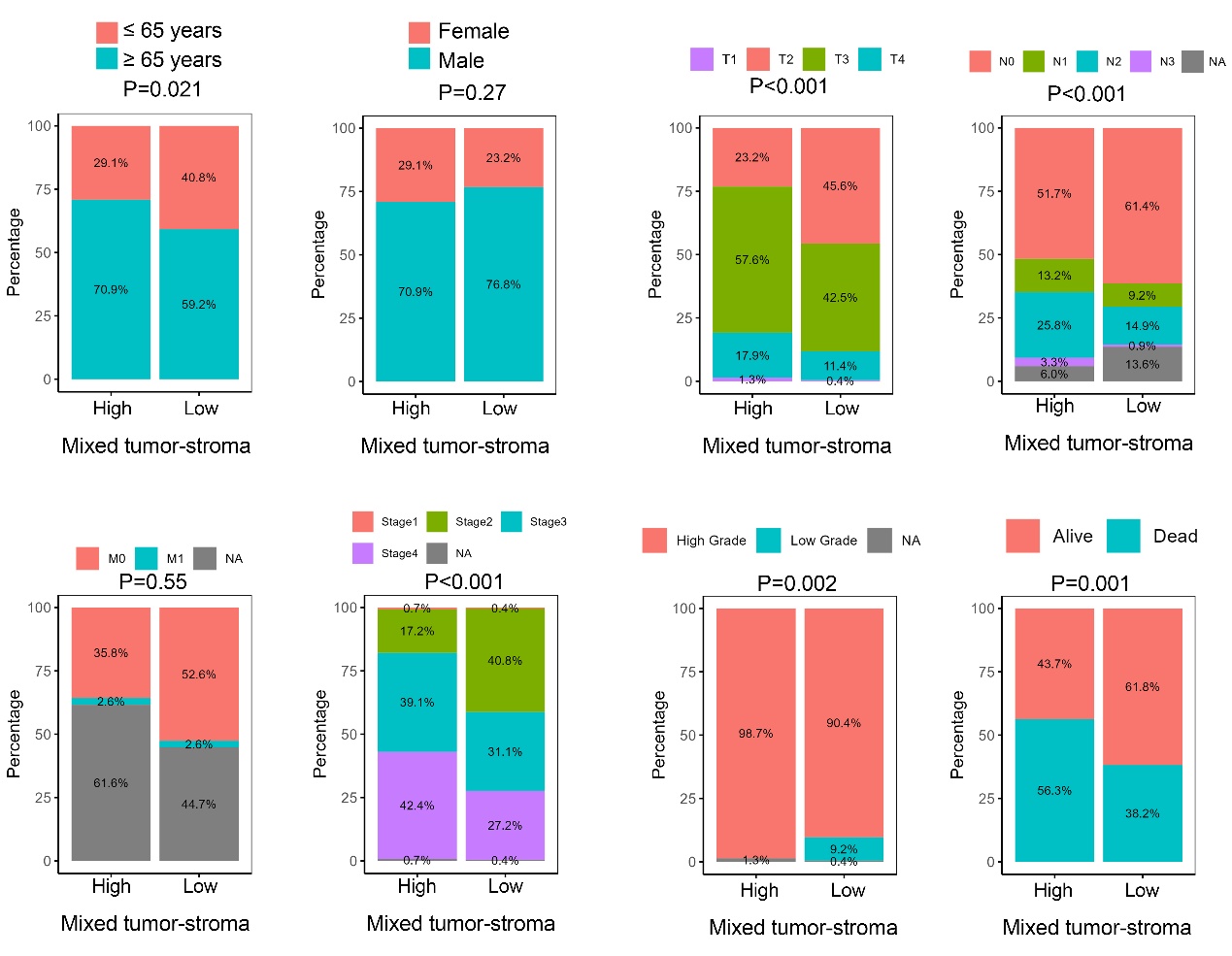


**Supplementary Figure 6** Clinical characteristics of high and low MTSR groups in TCGA.


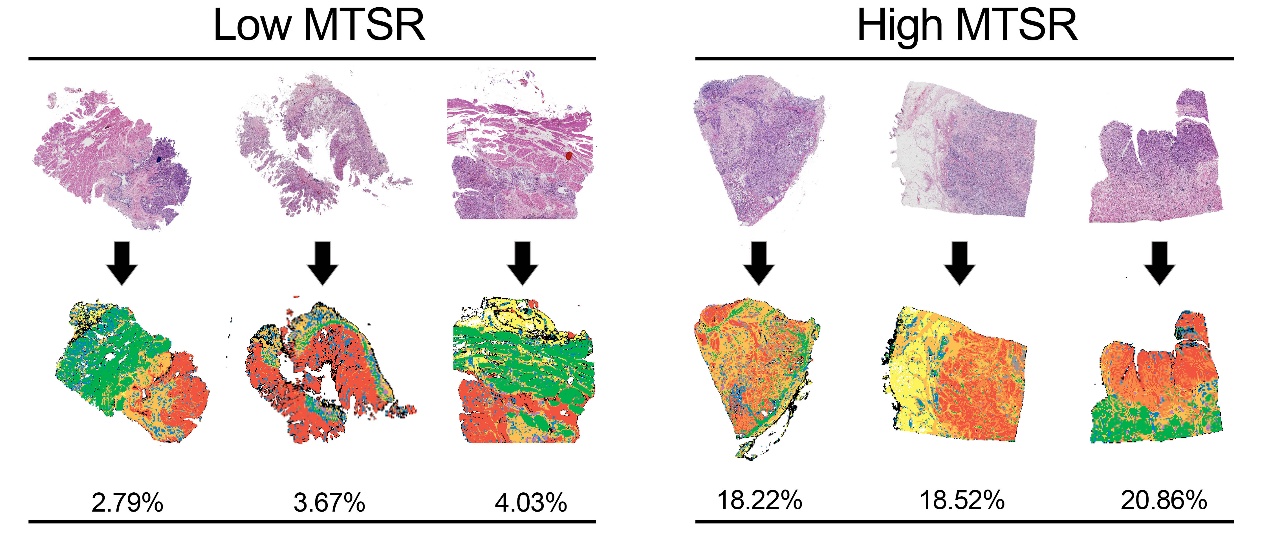


**Supplementary Figure 7** HE-stained WSI, histological subtype distribution, and MTSR values of BCa samples for single-cell sequencing.


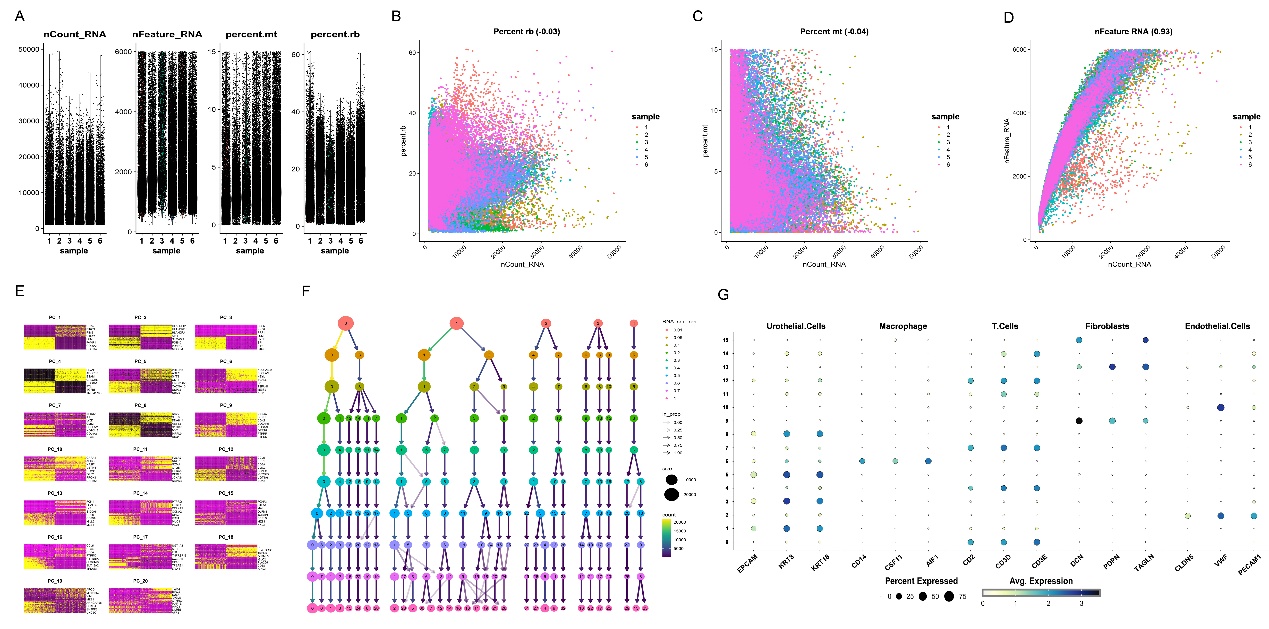


**Supplementary Figure 8** Key steps in the analysis of single-cell RNA-seq data. (A) Quality control plots: Distribution of RNA count, detected features, and percentages of ribosomal and mitochondrial genes across samples. (B–D) Distribution of RNA characteristics: Scatter plots showing the relationship between RNA count and ribosomal (B), mitochondrial (C), and gene counts (D). (E) Principal component analysis: Heatmaps of the top 20 principal components across cells. (F) Hierarchical clustering: Network plot showing relationships between samples based on RNA content and cell type proportions. (G) Cell type-specific gene expression: Dot plot showing gene expression across different cell types (Urothelial.Cells, Macrophage, T Cells, Fibroblasts, Endothelial Cells).


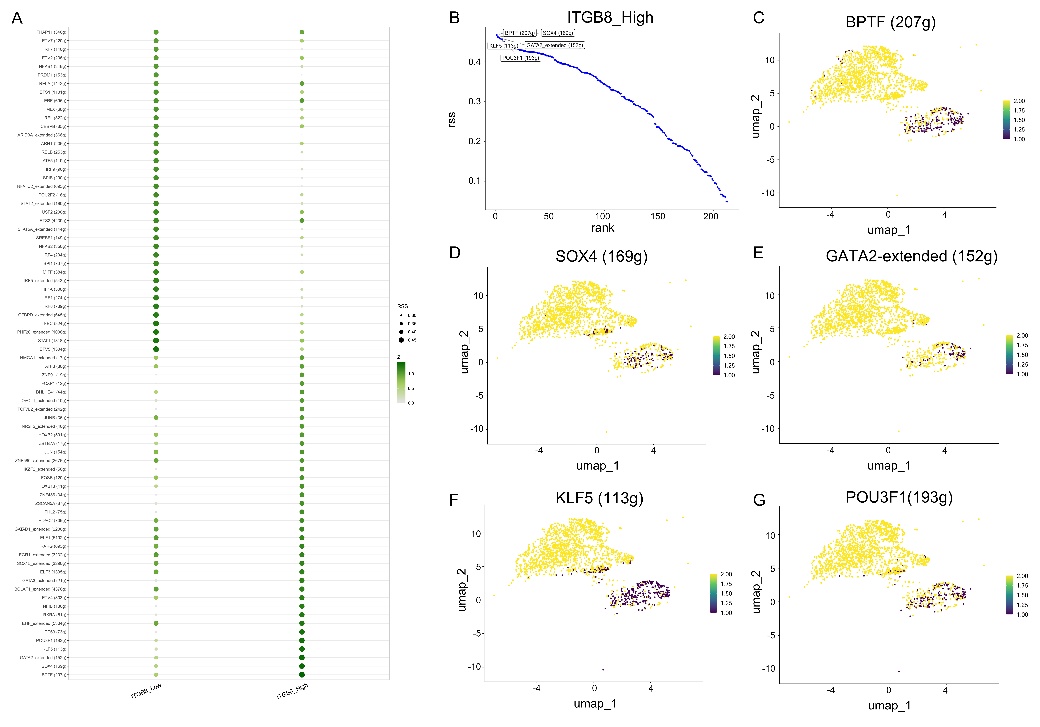


**Supplementary Figure 9** TF regulon landscape in ITGB8-low vs ITGB8-high cells(A) Bubble plot of SCENIC-inferred TF regulons across the ITGB8_low and ITGB8_high groups. Color encodes AUCell activity (Z-score) and dot size denotes the Regulon Specificity Score (RSS; computed from binarized AUCell), reflecting group specificity. Regulons were derived from GENIE3-inferred TF–target links combined with co-expression modules.(B) RSS ranking curve for the ITGB8_high group, highlighting the top regulons: BPTF, SOX4, GATA2-extended, KLF5, and POU3F1.(C–G) UMAPs showing per-cell AUCell activity of these five regulons (numbers in parentheses indicate the number of genes per regulon). Higher values indicate stronger activity.

**Supplementary Table 1** Top 10 radiomics features selected by mRMR.

DCE_log.sigma.3.0.mm.3D_firstorder_Maximum

DCE_wavelet.HHH_firstorder_Kurtosis

T2_wavelet.HHH_glszm_GrayLevelVariance

T2_wavelet.LLH_glrlm_LongRunLowGrayLevelEmphasis

T2_log.sigma.3.0.mm.3D_glcm_ClusterShade

DCE_log.sigma.2.0.mm.3D_glszm_LargeAreaLowGrayLevelEmphasis

DCE_wavelet.HLH_firstorder_Median

T2_log.sigma.2.0.mm.3D_glszm_SmallAreaLowGrayLevelEmphasis

T2_original_shape_Elongation

DCE_wavelet.LLH_glrlm_RunPercentage
